# Cell-Responsive Shape Memory Polymers

**DOI:** 10.1021/acsbiomaterials.2c00405

**Published:** 2022-06-10

**Authors:** Junjiang Chen, Lauren E. Hamilton, Patrick T. Mather, James H. Henderson

**Affiliations:** † BioInspired Syracuse: Institute for Material and Living Systems, Syracuse University, Syracuse, New York 13244, United States; ‡ Department of Biomedical and Chemical Engineering, Syracuse University, Syracuse, New York 13244, United States; § Department of Chemical Engineering, Penn State University, University Park, Pennsylvania 16802, United States

**Keywords:** shape-memory polymers, cell-responsive polymers, poly(ε-caprolactone), Pellethane, cytocompatibility

## Abstract

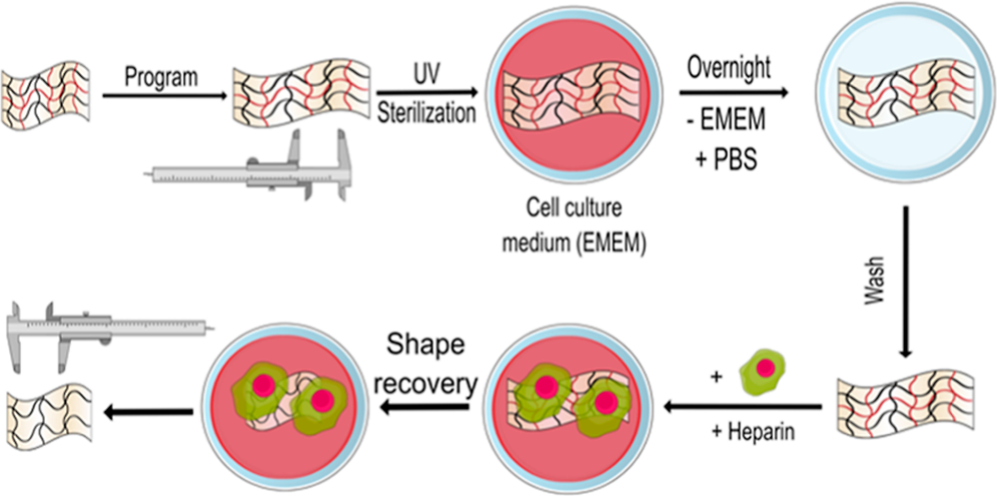

Recent decades have seen substantial interest in the development and application of biocompatible shape memory polymers (SMPs), a class of “smart materials” that can respond to external stimuli. Although many studies have used SMP platforms triggered by thermal or photothermal events to study cell mechanobiology, SMPs triggered by cell activity have not yet been demonstrated. In a previous work, we developed an SMP that can respond directly to enzymatic activity. Here, our goal was to build on that work by demonstrating enzymatic triggering of an SMP in response to the presence of enzyme-secreting human cells. To achieve this phenomenon, poly(ε-caprolactone) (PCL) and Pellethane were dual electrospun to form a fiber mat, where PCL acted as a shape-fixing component that is labile to lipase, an enzyme secreted by multiple cell types including HepG2 (human hepatic cancer) cells, and Pellethane acted as a shape memory component that is enzymatically stable. Cell-responsive shape memory performance and cytocompatibility were quantitatively and qualitatively analyzed by thermal analysis (thermal gravimetric analysis and differential scanning calorimetry), surface morphology analysis (scanning electron microscopy), and by incubation with HepG2 cells in the presence or absence of heparin (an anticoagulant drug present in the human liver that increases the secretion of hepatic lipase). The results characterize the shape-memory functionality of the material and demonstrate successful cell-responsive shape recovery with greater than 90% cell viability. Collectively, the results provide the first demonstration of a cytocompatible SMP responding to a trigger that is cellular in origin.

## 1 Introduction

Recent decades have seen substantial interest in the development and application of biocompatible shape-memory polymers (SMPs).^13^ SMPs have the ability to memorize a “permanent” shape through covalent or physical cross-linking.^1^ Fixation by crystallization, vitrification, or other means is then used to define the nonequilibrium “temporary” shape, which the SMP will maintain until a triggering event, such as thermal,^2^ light,^3^ or solvent^4^ exposure, is applied, and the SMP recovers to the permanent shape. In 2002, the first biocompatible SMP was reported by Lendlein and Langer, with the motivating application being smart biodegradable sutures.^5^ Subsequent work on biocompatible SMPs included SMPs designed for drug delivery,^6^ treatment of vascular disease,^7^ bone tissue engineering,^8^ antibacterial functionality,^9^ and a host of other applications^10−12^ in which both rapid^13^ and prolonged recoveries^14,15^ have been demonstrated.

As shape-memory materials science has advanced, efforts have been sought to develop SMPs that are not only biocompatible but also cytocompatible and can be triggered safely without harming the cells present in vitro (e.g., cell culture-compatible) or in vivo.^16,17^ Initial works on cytocompatible SMP triggering by our group and others focused on thermal triggers via changes in ambient temperature.^18−20^ In light of the potential for ambient temperature changes to affect cell biological processes being studied in vitro or to damage surrounding tissues or organs in vivo, stimuli that do not require a change in ambient temperature have since been studied.^21^ For instance, solvent-responsive SMPs are a class of SMPs in which an amorphous or semicrystalline network can be plasticized by small solvent molecules.^22^ Similarly, the hydrogen bonds in polymer networks can also be weakened by bound water, with the weakening of the hydrogen bond between N–H and C=O groups^23^ and the dissociation or disruption of the hydrogen bond^24^ reducing the flexibility of the chains, thus decreasing the glass-transition temperature (*T*_g_). Photothermal (light-based) triggering is another approach studied to tackle this issue.^25^ As a stimulus, light presents a number of potential advantages, such as the avoidance of undesired heating in surrounding media or tissues during actuation;^26^ remote activation tunable via the control of wavelength, light intensity, and direction, thereby enabling triggering through intervening media or tissues;^27^ and high-resolution spatial control of the recovery.^28^

Although prior work has used SMP platforms triggered by thermal,^29,30^ solvent,^31,32^ or photothermal^33^ events to study cell mechanobiology, SMPs triggered directly by cell activity have not previously been demonstrated. SMPs that can be triggered directly by cell activity could enable new lines of biomaterials science research and new biomedical strategies, such as drug delivery vehicles that target specific cells or organs and decision-making biosensors to control patient treatment. As an enabling step toward the demonstration of a cell-responsive SMP, we previously^34^ developed an SMP that can respond to enzymatic activity at the cell culture (human body) temperature. The enzymatically (lipase) triggered SMP is prepared by dual electrospinning of poly(ε-caprolactone) (PCL) and a thermoplastic polyurethane, Pellethane. PCL acts as a shape-fixing agent, while Pellethane acts as a shape-recovery agent, as PCL is an enzymatically labile fixing component and Pellethane is an enzymatically stable polyether-based thermoplastic polyurethane elastomer. After fixation, the PCL portion of the SMP is under compression and the Pellethane portion under tension. Lipase cleaves the ester bonds in the PCL network, leading to the eventual degradation of PCL, and the contraction of Pellethane drives the material back to its original shape. Although successful in achieving enzyme responsiveness, the previous study only demonstrated triggering in response to artificially prescribed enzyme concentrations with no cells present.

**Scheme 1:**
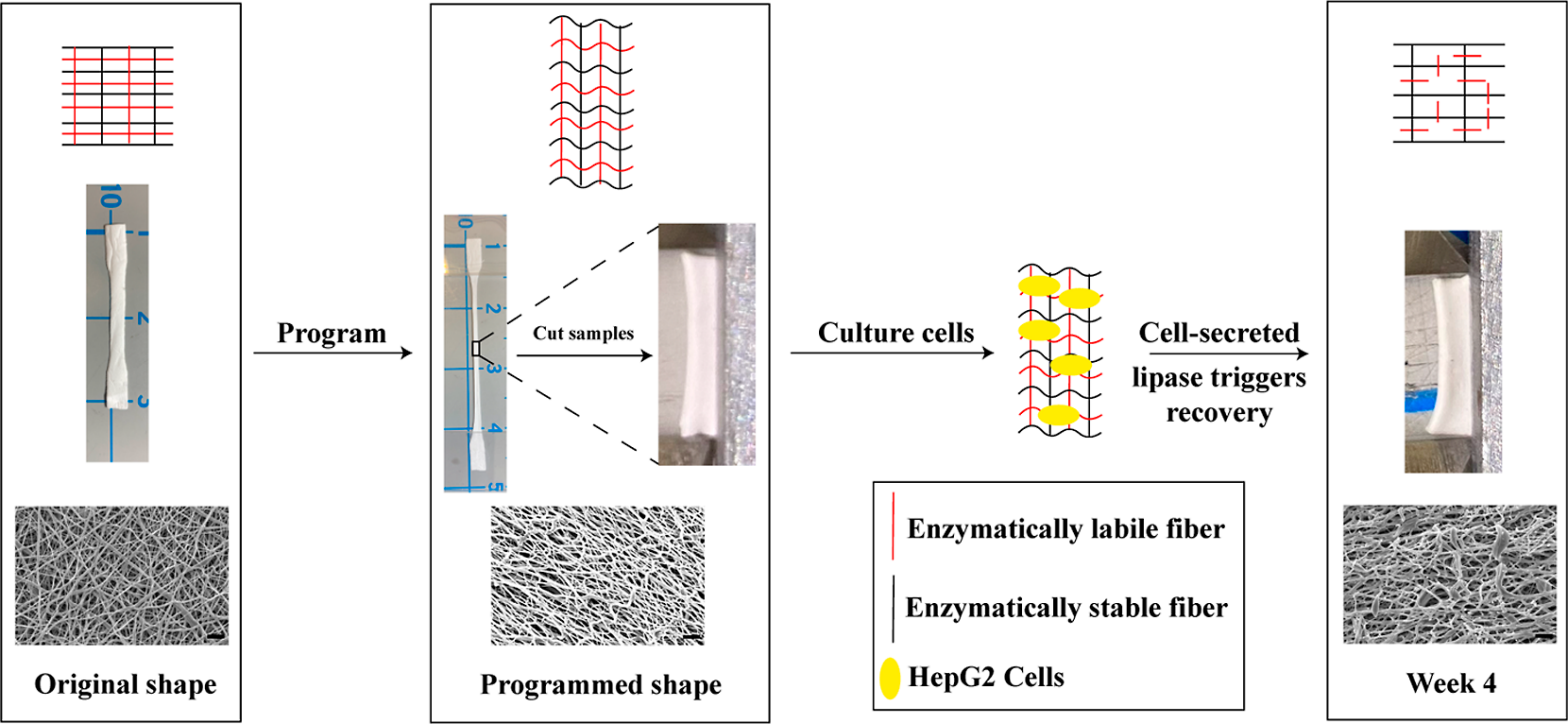
Approach for Demonstrating Enzymatic Triggering of a Shape-Memory Polymer in Direct Response to the Presence of Enzyme-Secreting Human Cells Sample from the PCL–PEL 400 group (text for details), with the illustration of the mechanism (top row), and macro- (middle) and micro- (bottom) views as the sample undergoes the shape-memory cycle.

Here, our goal was to build on that enabling work by demonstrating the enzymatic triggering of an SMP in direct response to the presence of enzyme-secreting human cells. To achieve this goal, we studied the extent to which HepG2 cells, a human liver cancer cell line that expresses hepatic triglyceride lipase activity, could trigger shape-memory activation of a scaffold tailored for cell triggering (Scheme 1). PCL–Pellethane SMP samples were fabricated by electrospinning; thermal properties were analyzed by thermogravimetric analysis (TGA) and differential scanning calorimetry (DSC); surface morphology was analyzed by scanning electron microscopy (SEM); and shape-memory performance over a 4 week period and cytocompatibility over a 1 week period were quantitatively and qualitatively analyzed.

## 2 Materials and Methods

### 2.1 Materials

The Lubrizol Corporation kindly supplied Pellethane 5863-80A (hereafter referred to as “Pellethane”) pellets. Tetrahydrofuran (THF) was purchased from VWR International. *N*,*N*-Dimethylformamide (DMF), chloroform (CHCl_3_), and PCL (*M*_n_ = 80,000 g/mol) pellets were purchased from Sigma-Aldrich. Heparin, Triton X-100, *p*-nitrophenyl palmitate, and Dulbecco’s phosphate-buffered saline (PBS) were purchased from Fisher Scientific. HepG2 cells, Eagle’s minimum essential medium (EMEM), and penicillin/streptomycin were obtained from the American Type Culture Collection (ATCC). Fetal bovine serum (FBS) and Live/Dead stain were obtained from Invitrogen.

### 2.2 Study Design

SMP samples were fabricated by dual-jet electrospinning of PCL and Pellethene, as previously described.^34^ Samples were incubated for 4 weeks in six-well tissue culture plates, with HepG2 cells cultured directly on the samples by suspension seeding and with or without the addition of 3 μg/mL heparin, the presence of which increases the secretion of hepatic lipase, with a culture medium change every week. Samples containing only PCL were used as a control that had no enzymatically stable agent, and samples containing only Pellethane were used as a control that had no enzymatically labile agent. TGA, DSC, SEM, and length measurements were used for quantitatively and qualitatively assessing the thermal properties, change in microstructure morphology, and change in length during the recovery process. Live/Dead assay was used to analyze the cytocompatibility of the fibers with respect to cells cultured on the surrounding tissue culture plate (a noncontact, indirect cytocompatibility assay).

### 2.3 Fabrication

Samples containing 20% PCL were fabricated, as this PCL composition provided the highest cytocompatibility in our prior work.^34^ Following the previous protocol, an 11 wt % Pellethane electrospinning solution was prepared by dissolving Pellethane in a solution of DMF and THF at a ratio of 1:1.5 by volume.^34^ A 15 wt % PCL electrospinning solution was prepared by dissolving PCL in a solution of DMF and CHCl_3_ at a ratio of 1:4 by volume. The solutions were continuously stirred until the polymers were dissolved completely.

All samples were fabricated by dual-jet electrospinning using a custom electrospinning apparatus composed of a rotating cylindrical drum collector (5 cm diameter), a Spraybase electrospinning syringe pump, a Thermofisher electrospinning syringe pump, a high-voltage positive power supply (Agilent E3630A), and a low-voltage negative power supply (PS 500XT, Hoefer Scientific). Fiber mats of 20 wt % PCL were fabricated by setting the flow rate of PCL at 0.5 mL/h with a total delivered volume of 3.6 mL, while the Pellethane flow rate was set at 2 mL/h with a total delivered volume of 22 mL, with the longer duration of Pellethane delivery resulting in a fiber mat of PCL covered by Pellethane. 22 G needles were used, and 12 and 13.6 kV voltages were applied to the PCL and Pellethane needle tips, respectively, with a needle-to-mandrel distance of 10 cm. A negative voltage of −500 V was applied to the mandrel to improve the fiber deposition. Rotational speeds of 400 and 3000 rpm were used to create random (PCL 400, PEL 400, and PCL–PEL 400) and aligned (PCL 3000, PEL 3000, and PCL–PEL 3000) fibers, respectively, to study the effect of fiber organization on recovery. Controls were fabricated similarly but with only one needle to deliver a single polymer.

### 2.4 Thermal Analysis

Thermal degradation of all samples was measured by TGA (TA Instruments Q500). When an increase in mass loss rate was detected, the heating rate was decreased to achieve a high-resolution analysis of thermal degradation activities (TA Instruments Dynamic Rate Hi-Res Ramp). All samples were heated to 600 °C at a maximum rate of 50 °C/min. A resolution of 4 °C and a sensitivity value of 1 were used, as previously described.^34^

DSC (TA Instruments Q200) was performed to analyze thermal transitions and calculate fiber compositions for a portion of each fiber mat.^34^ Briefly, 3–5 mg samples were loaded into a T-zero aluminum pan for each test. Each fiber mat was cooled and equilibrated at −60 °C to erase any thermal history, heated at 10 °C/min to 170 °C and then immediately cooled at 5 °C/min to −60 °C. *T*_g_ and the melting transition temperature (*T*_m_) were measured by heating the samples again at 10 °C/min to 170 °C. The heat of crystallization of PCL was used to estimate the composition of each fibrous web via eq 1.
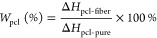
1

Only fiber mats having a composition value of 20% (±5%) were used in the subsequent experiments.

### 2.5 SEM Imaging

SEM (JEOL 5600) was used to observe the changes in microstructure morphology in the fiber mats. As-spun, programmed, and cell-triggered samples were imaged. All samples were mounted, sputter-coated with Au for 45 s (Denton Vacuum-Desk II), and imaged with an accelerating voltage of 10 kV.

### 2.6 Cell-Triggered Shape Recovery Experiments

HepG2 cells were used to study cell-triggered shape recovery. Cells were cultured in EMEM, supplemented with 10% FBS, 1% penicillin/streptomycin, and with or without 3 μg/mL heparin, a concentration within the normal physiological range of human plasma (1–5 μg/mL).^35^ EMEM without phenol red was used when analyzing the lipase concentration in the extracellular medium to minimize the influence of phenol red on the absorbance measurement.^36^ Cells were cultured under 37 °C with 5% CO_2_.

Cell-triggered shape recovery was assessed by culturing HepG2 cells directly on the samples by suspension seeding in six-well tissue culture plates for 4 weeks, with a seeding density of 10^5^ cells/well and a cell culture medium volume of 3 mL/well. Prior to culture, the samples were cut into a dog bone shape (ASTM D638 type IV, scaled down by a factor of 4), heated, and stretched to 300% strain (aligned samples were stretched in the direction perpendicular to the fiber alignment direction) using a custom, screw-driven manual stretcher. Samples consisting of programmed pure PCL fibers are not reported, as mechanical stretching of the thin PCL fibers of the control caused yield during the programming process. The stretched samples were unloaded from the stretcher after cooling in a −20 °C freezer for 10 min. The programmed samples were cut into 1 cm long pieces. The length of each sample piece was measured by calipers and photographed before and after culturing with cells. For shape recovery and indirect cytocompatibility test, all samples were soaked in complete medium overnight to allow proteins to adsorb throughout the samples before cell seeding. Samples were washed with PBS and transferred into new tissue culture plates before seeding. For recovery, cells were added to the tissue culture plates with samples present, resulting in cell attachment in the fiber structure and on the tissue culture plate. Experiments were conducted over 4 weeks, with one sample collected for analysis every 7 d. Upon collection, the samples were washed using deionized water and dried in a vacuum oven for 72 h at room temperature. Every experiment was repeated three times, for *n* = 3 replicates.

### 2.7 Indirect Cytocompatibility

Indirect cytocompatibility of the samples was analyzed by Live/Dead assay. Cells were seeded on 24-well tissue culture plates, and then samples were added to the wells. The cells were cultured for 1 week without medium change. The initial seeding density was 5 × 10^4^ cells/well, with a total medium volume of 1 mL/well. After culturing the samples with cells for 24 h (day 1), 72 h (day 3), 120 h (day 5), and 168 h (day 7), the samples were removed, and the tissue culture well plates were washed and stained using Live/Dead assay. Live cells were labeled by green fluorescent calcein-AM, which detects the intracellular esterase activity. Dead cells were labeled by red fluorescent ethidium homodimer-1 (EthD-1), which detects the loss of plasma membrane integrity. Cells growing on the tissue culture well plates without samples were used as the live control. The dead control was prepared by removing the cell culture medium 20 min before staining, washing with PBS, and adding 70% ethanol. Cells were detached by 0.25% trypsin–ethylenediaminetetraacetic acid solution, and the total cell number was counted manually using a hemocytometer. Cell viability was determined as the percentage of live stained cells (those not presenting dead stain) among the total cells counted.

### 2.8 Lipase Activity Detection

Both extracellular and intracellular lipase concentrations were assayed at days 1 and 7, with *p*-nitrophenyl palmitate used as the chemical substrate for the measurements.^37^ As an additional step, cell lysis, was needed for the intracellular assay, two different standard curves were used in the extracellular (Figure S1A) and intracellular calculations (Figure S1B).

For the extracellular lipase concentration assay, solution A was prepared by adding 100 μL of cell culture medium to 100 mM Tris-HCl buffer (pH 8.0), with 1%V/V Triton X-100, as nitrophenyl palmitate is not soluble in this buffer. Solution B was 5 mM *p*-nitrophenyl palmitate solution in acetonitrile. Solution A was incubated at 45 °C with stirring for 10 min. Then, 2 mL of solution A was added to 50 μL of solution B. Lipase activity was determined by monitoring the absorbance at 405 nm. The cell culture medium with no cells was used as the control. The standard curve for optical density (OD) versus lipase concentration measured the absorbance at several known lipase concentrations. Linear regression was performed to fit the standard curve (Figure S1A) for lipase concentrations below 5 μg/mL: OD = 68.981*c* (*R*^2^ = 0.9483), where *c* is the lipase concentration.

For the intracellular lipase concentration assay, cells were seeded on 96-well plates with an initial seeding density of 5000 cells/well and cultured for 1,3, 5, and 7 d; cell numbers of a second 96-well plate with the same culturing conditions were counted using a hemocytometer; and the total cell volume was calculated as total cell number times average single-cell volume (850 μm^3^).^38^ The cells were washed twice with PBS and lysed in 50 μL of 1% V/V Triton X-100 in PBS at 4 °C overnight. 50 μL of 100 mM Tris-HCl buffer (pH 8.0), with 1%V/V Triton X-100, was added to each well and incubated at 45 °C for 10 min with shaking; then, 20 μL of solution B was added; and the absorbance at 405 nm was measured. The linear equation can be applied to the standard curve (Figure S1B): OD = 161.684*c* (*R*^2^ = 0.944).

Intracellular and extracellular lipase concentrations were calculated based on the above standard curves. For the intracellular lipase concentration calculation, the total lipase amount was divided by the total cell volume.

### 2.9 Statistical Methods

All experiments were repeated three times (*n* = 3). One-way ANOVA, followed by Tukey’s post hoc test between groups or two-way ANOVA, followed by Holm–Sidak’s multiple comparisons test between groups was performed, as indicated. Significance was set at *p* < 0.05 (*p < 0.05, **p < 0.01). Results are reported as mean ± standard deviation.

## 3 Results

### 3.1 TG and DSC Analyses

**Figure 1:**
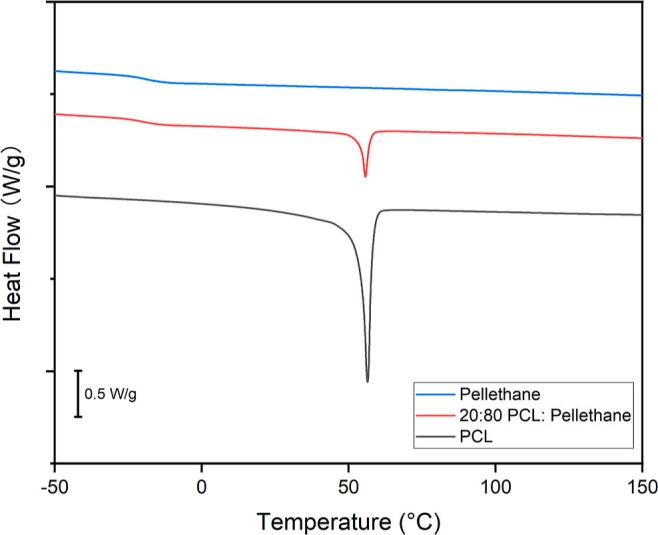
Content percentage was calculated via DSC by dividing the enthalpy change of PCL (approximately 41.5 J/g) by the enthalpy change of the PCL–Pellethane fiber (approximately 8.5 J/g). The calculated composition of the representative sample being shown is 20.48%. Only content percentages close (±5%) to the predicted values were used in subsequent experiments.

Thermal analysis was conducted to confirm the composition and phase transition. Consistent with our previously reported findings,^34^ TGA showed that the as-processed fiber composites comprise separate phases (Figure S2). Because the degradation of PCL and Pellethane was superimposed in the composites, it was challenging to analyze the content percentage of the PCL–Pellethane fibers. As such, the content percentage was analyzed by DSC (Figure 1). DSC analysis showed a *T*_g_ of Pellethane of approximately −19 °C and *T*_m_ of PCL of approximately 56 °C. The content percentage^39^ was estimated by dividing the enthalpy change of PCL (approximately 41.5 J/g) by the enthalpy change of the PCL–Pellethane fibrous composite (approximately 8.5 J/g). Only content percentages close (±5%) to the predicted values were used in subsequent experiments. It should be noted that this estimation assumes that the degree of crystallinity was the same in the composite and pure PCL webs.

### 3.2 Lipase Concentration

**Figure 2:**
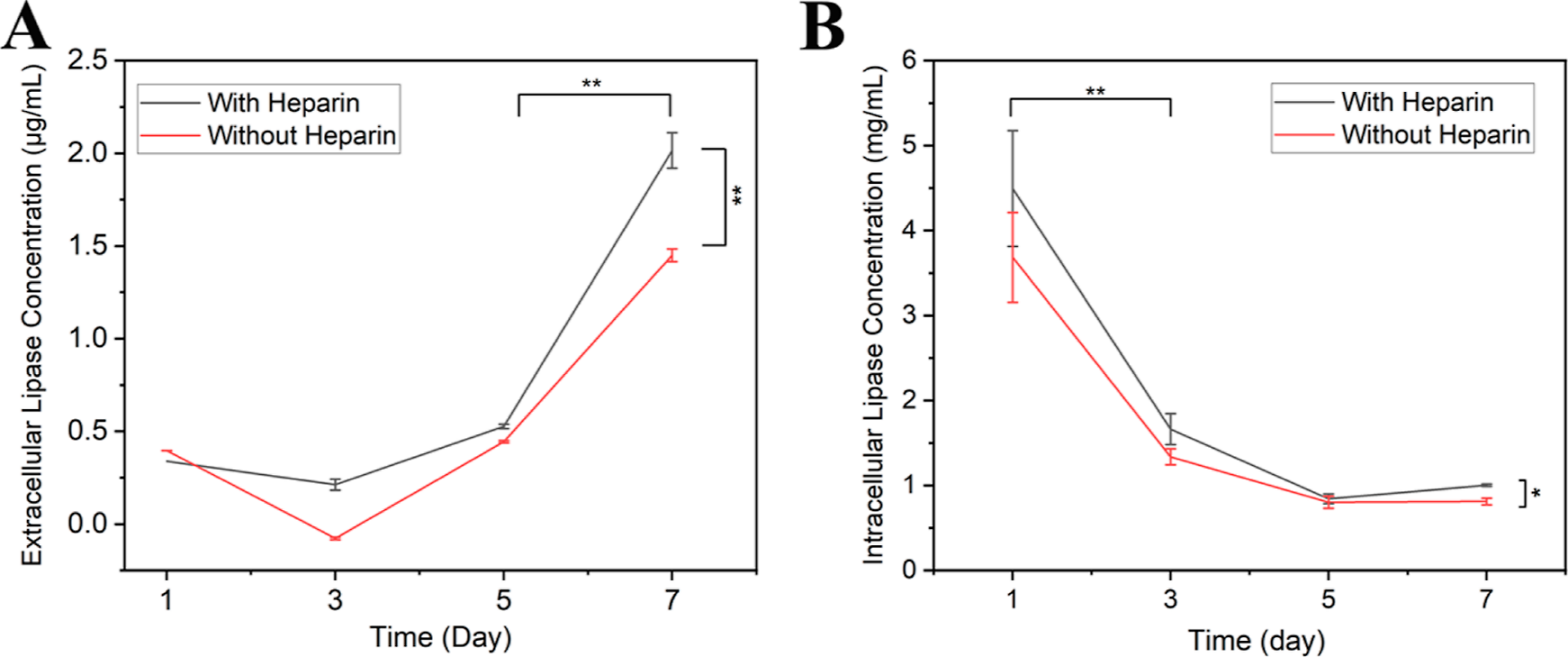
(A) Extracellular lipase concentration in the medium of cultured HepG2 cells increased over time, regardless of whether or not the cells were treated with heparin, but, by day 7, cells treated with heparin had a significantly higher lipase concentration than cells without heparin treatment. (B) Intracellular lipase concentration of the cultured HepG2 cells decreased with time but was orders of magnitude higher than the extracellular lipase concentration. The relation between absorbance and lipase concentration can be found in the Supporting Information (Figure S1A,B) (*n* = 3, two-way ANOVA, followed by Holm–Sidak’s multiple comparisons test between groups. *p < 0.05, **p < 0.01).

The extracellular lipase concentration was found to increase with the increasing incubation time, regardless of whether the cells were treated with heparin (Figure 2A), but, by day 7, cells treated with heparin had a significantly higher lipase concentration (2.02 ± 0.09 μg/mL) than cells without heparin treatment (1.45 ± 0.03 μg/mL). The increase in the extracellular lipase concentration and its timing proved important to the cell-triggered shape-memory phenomenon, as described below.

In contrast, the intracellular lipase concentration decreased between day 1 and day 7 (Figure 2B) for both heparin-treated cells (decrease from 4.50 to 1.00 mg/mL) and cells without heparin treatment (decrease from 3.69 to 0.81 mg/mL).

In previous acellular experiments, lipase concentrations above 0.5 mg/mL were required for shape recovery.^34^ Despite the highest extracellular lipase concentration in the present work being below 0.5 mg/mL and in the μg/mL range, intracellular lipase concentrations were orders of magnitude higher, in the mg/mL range, and consistent above 0.5 mg/mL, providing a mechanism whereby the HepG2 cells could trigger shape recovery via elevated enzyme concentration in the vicinity of the cells.

### 3.3 Scanning Electron Microscopy

All 400 rpm as-spun fibers showed a random orientation, while 3000 rpm ones showed an aligned orientation (Figure S3). All fibers exhibited a bead-free morphology. After programming, the relatively simple paths of the highly aligned PCL–PEL fibers became more tortuous and without preferential alignment (Figures S8f and S9f), a result of tensile strain being stored in the stretching direction with the fiber network under tension. A looser arrangement was found in the PEL 3000 fibers (Figure S7f), while no significant changes were seen in the PEL 400 fibers (Figure S6f).

**Figure 3:**
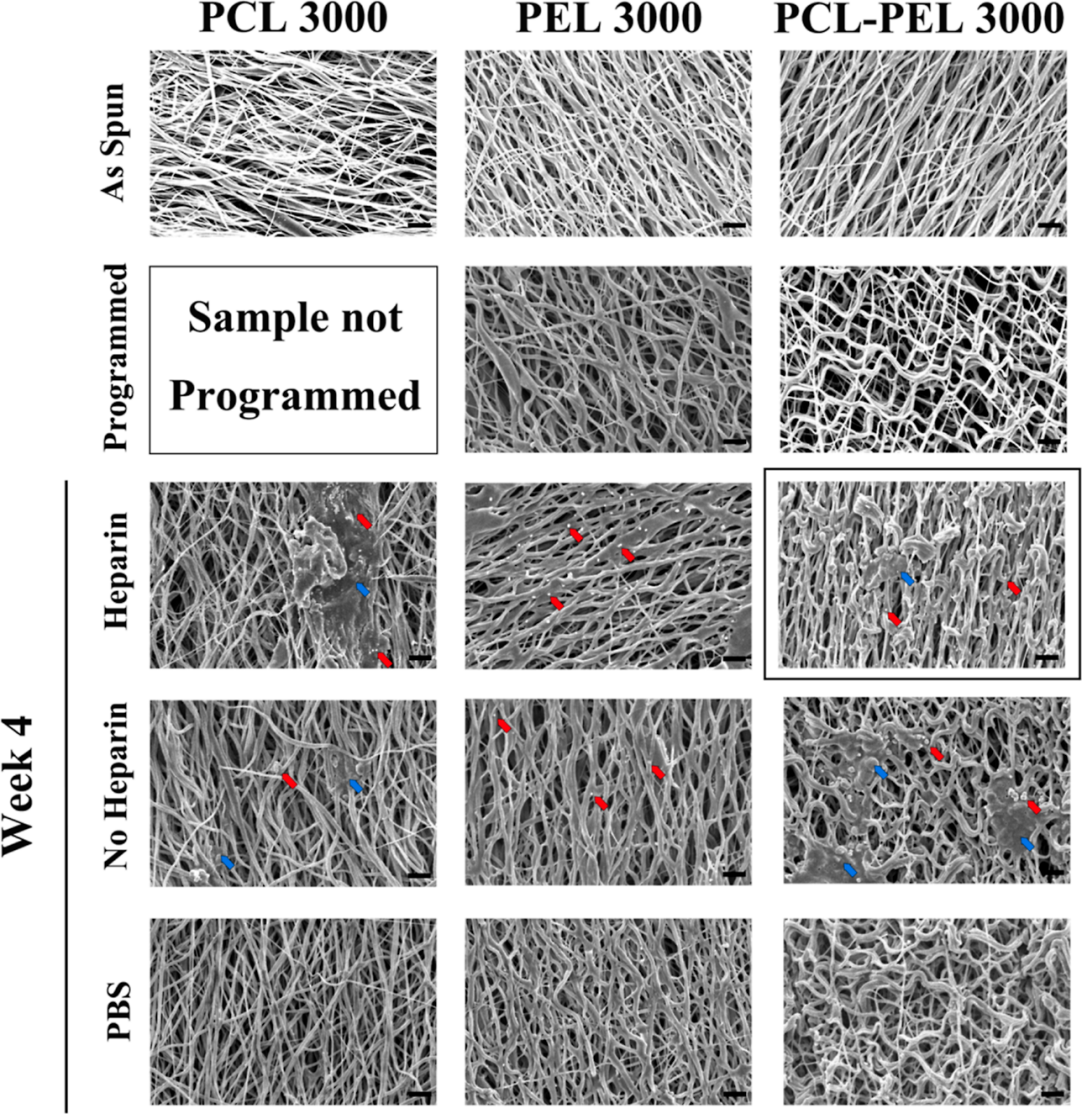
Presence of heparin-treated cells led to recovery of the fiber structure to the as-spun state after 4 weeks of incubation (boxed image), while no significant recovery-related morphological change was found in cells without heparin treatment or in samples in PBS. PCL 3000 fibers showed no recovery-related morphological changes after 4 weeks of culture in PBS but became coarser over time and eventually showed a film-like morphology at 4 weeks in some areas of the fiber networks in the presence of cells. PEL fibers showed no morphological changes after 4 weeks of culture under any control or treatment conditions compared to the programmed ones. Unidentified small particles or binders (representative particles or binders are identified by arrows) were observed to be embedded in the fiber networks in the presence of cells. All 3000 rpm as-spun fibers showed an aligned orientation. The programmed PEL 3000 and PCL–PEL 3000 showed a more tortuous arrangement without preferential alignment compared to the as-spun samples. Representative particles (red) or binders (blue) are identified by arrows. Scale bar: 10 μm.

The key finding of the SEM imaging analysis was that the presence of heparin-treated cells led to the recovery of the fiber structure to the as-spun state (see Figure 3 for the representative aligned PCL 3000, PEL 3000, and PCL-PEL 3000 samples and Figure S3 for the representative samples from all groups). In contrast, neither random PCL 400 (Figure S4) nor aligned PCL 3000 (Figure S5) fibers showed morphological changes after 4 weeks of culture with (control) PBS. Similarly, culture with non-heparin-treated cells resulted in no significant recovery-related change in fiber morphology at 4 weeks.

Accumulating discontinuities in fibers in both PCL-PEL 400 and PCL-PEL 3000 samples (Figures 3, S8 and S9) provide evidence of degradation of PCL over the culture period. Discontinuities in both fiber types were found to begin within the first week after culture with heparin-treated cells, indicative of the initiation of degradation of PCL. Particles or binders (representative particles or binders are identified by arrows in Figures 3, S8, and S9) were observed in both PCL-PEL 400 and PCL-PEL 3000 fibers after being cultured with cells. Coarse fibers were observed in both sample types, following incubation in PBS or with non-heparin-treated cells for 4 W, which could be evidence of “melting” of PCL despite the ambient temperature being below *T*_m_ for polymeric (non-oligomeric) PCL. This was consistent with expectations, as the morphology of PCL would be anticipated to change over time when cultured with enzyme-secreting cells, as PCL is an enzymatically labile agent, which would respond to the enzymatic activity. In contrast, neither PEL 400 (Figure S6) nor PEL 3000 (Figure S7) fibers showed morphological changes after 4 weeks under any control or treatment conditions. This was also consistent with expectations, as Pellethane is an enzymatically stable agent, which would not respond to the enzymatic activity. After 4 weeks of incubation with heparin-treated cells, PCL-PEL 3000 fibers returned to a denser linear structure compared to the programmed ones. These morphology changes are indicative of a cell-triggered shape recovery phenomenon. Moreover, compared to the as-spun samples, after week 4, both PCL-PEL 400 and 3000 fibers showed a similar but looser morphology with more binders and discontinuities in the microstructure.

### 3.4 Cell-Triggered Shape Recovery

**Figure 4:**
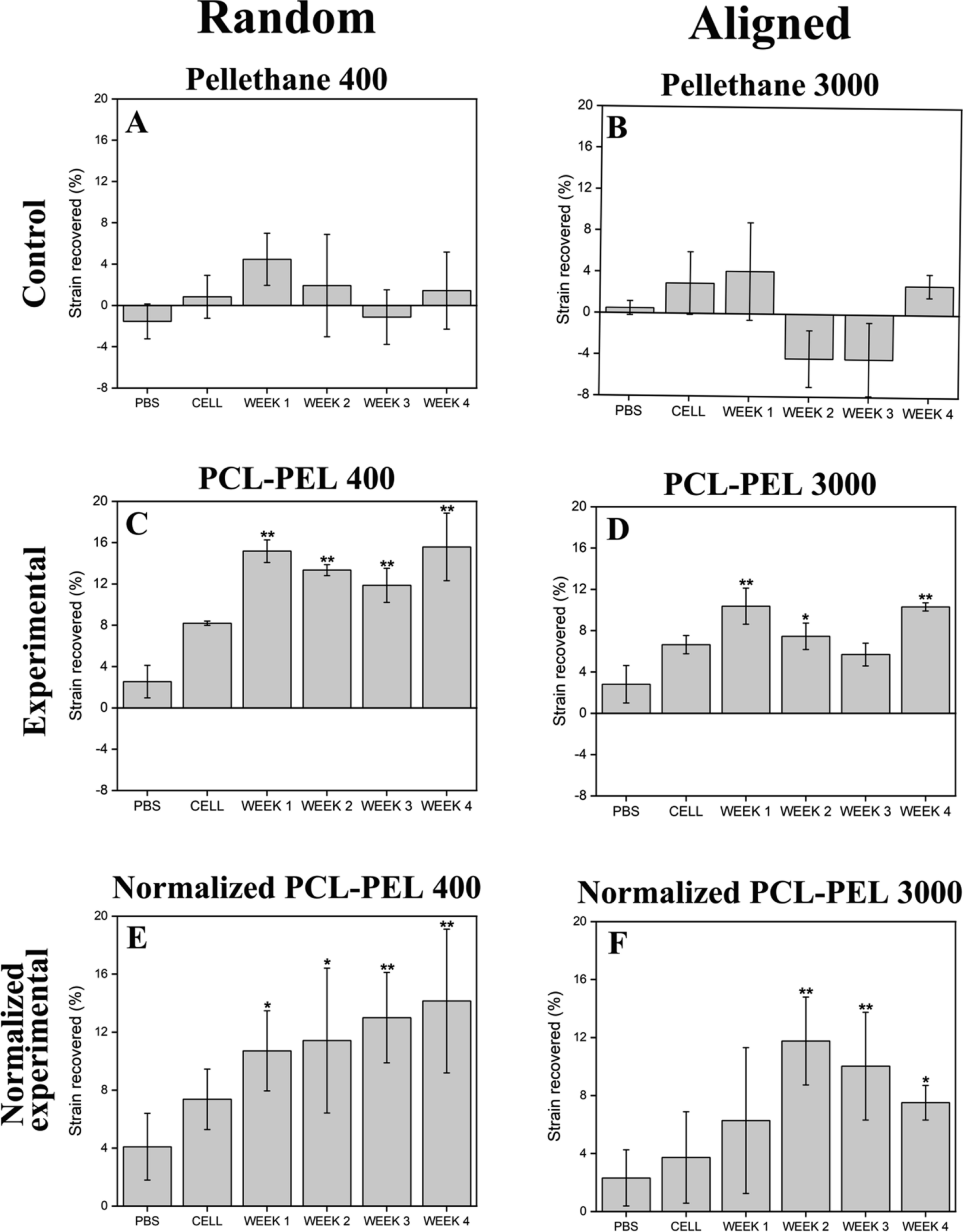
Shape recovery was successfully triggered by heparin-treated cells in random and aligned PCL–PEL fibers. Strain recovered during shape recovery over 4 weeks was calculated. To account for the non-shape-memory changes, such as curling, observed in the Pellethane controls, the strain change of the Pellethane controls (Figure 3A,B) was subtracted from that of the PCL–PEL experimental groups (Figure 3C,D) to determine the normalized shape-memory recovery (Figure 3E,F). The majority of strain recovery was completed by 2 weeks of incubation. Trends without statistical significance were observed in the strain change of the control groups: PBS and cells without heparin treatment (PBS and CELL; *n* = 3). One-way ANOVA was followed by Tukey’s post hoc test between groups. Markers indicating a significant difference (*p < 0.05, **p < 0.01) are for comparisons made against the PBS control.

Shape recovery was successfully triggered by heparin-treated cells in random and aligned PCL–PEL fibers (Figures 4C–F and S10). The majority of strain recovery was completed within the first 2 weeks of incubation, after which no further statistically significant change in length was observed over the 4 week experiment. In contrast, no significant differences in the strain change were observed in control groups, namely PBS and cells without heparin treatment, for 4 weeks (PBS and CELL groups in Figure 4). In addition, random-oriented fibers were found to recover more than aligned fibers (Figure 4C vs 4D, and 4E vs 4F). Both the random (Pellethane 400) and aligned (Pellethane 3000) Pellethane control groups showed similar, nonsignificant trends in shape change. These changes in shape were due not to the shape-memory effect but to other changes in shape that occurred during treatment, such as sample curling, which complicated the precise measurement of sample dimensions. To account for these non-shape-memory changes, normalization was performed (Figure 4E,F) by subtracting the strain change of the Pellethane controls (Figure 4A,B) from that of the PCL–PEL experimental groups (Figure 4C,D).

### 3.5 Indirect Cytocompatibility

**Figure 5:**
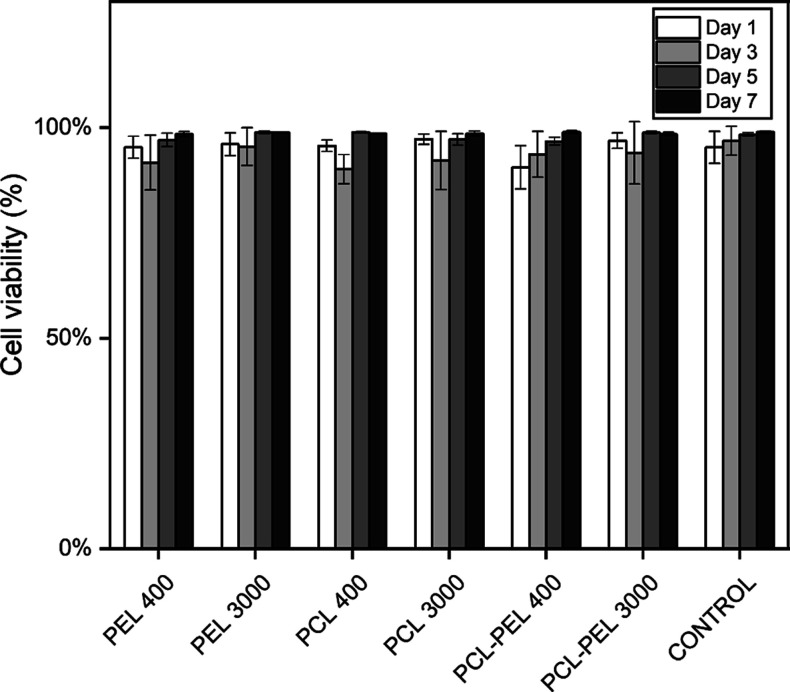
Analysis of cell viability of HepG2 cells cultured on tissue culture plates with fiber composites for 7 d found no significant statistical differences in cell viability compared to control groups. All groups had a viability of 90% or greater over 7 d (*n* = 3, one-way ANOVA, followed by Tukey’s post hoc test between groups).

**Figure 6:**
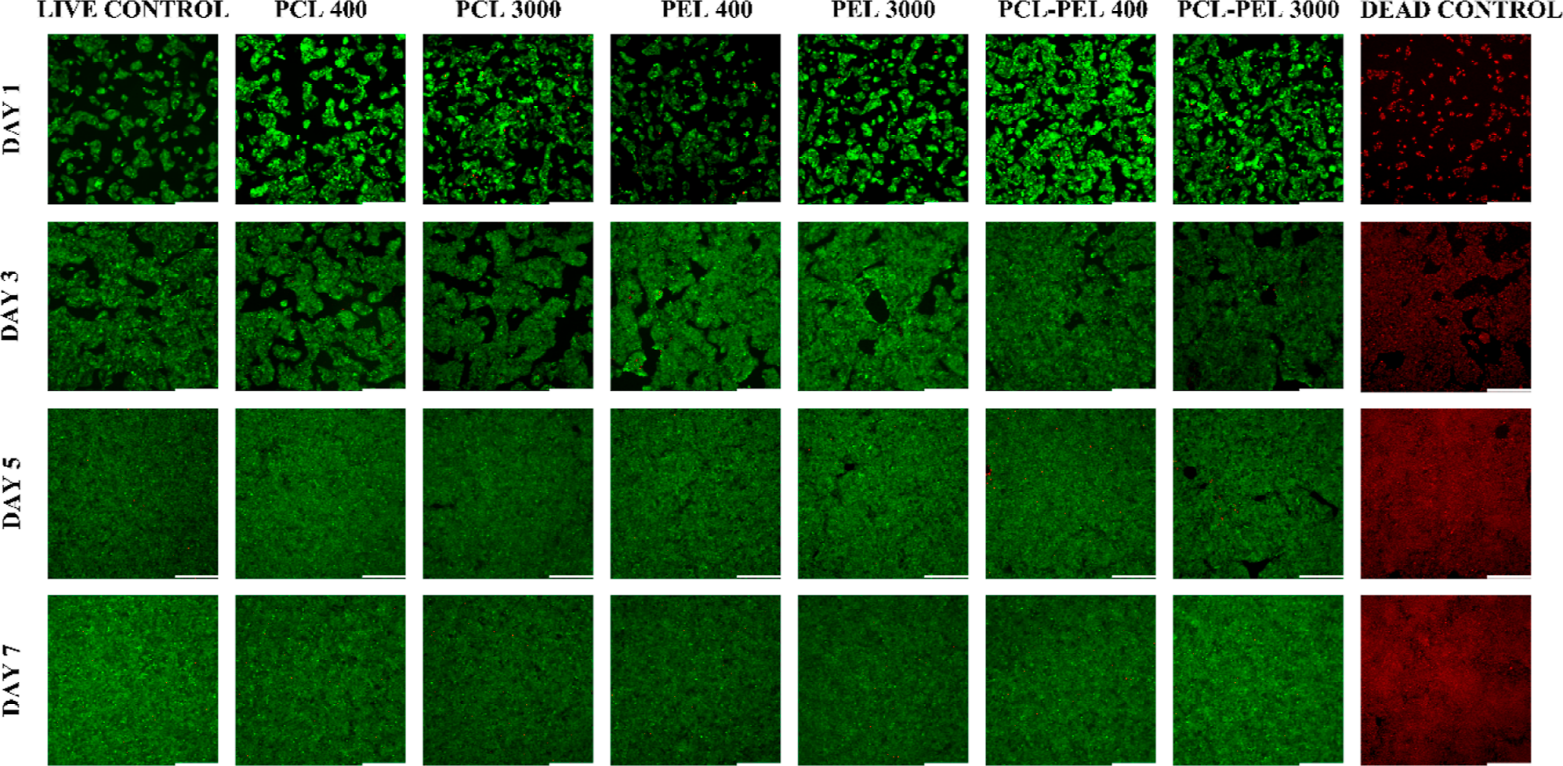
Live/dead micrographs of HepG2 cells cultured on tissue culture plates with fiber composite and noncomposite control showed cell proliferation over the period of incubation and few dead cells (red dots) in all groups, which revealed the indirect cytocompatibility of the fibers. Scale bar: 330 μm.

The results of the Live/Dead assay showed that the fibers are cytocompatible when cultured with HepG2 cells over 7 d. No significant statistical differences in cell viability were found (Figure 5), as compared to control groups. All groups had a viability of 90% or greater through 7 d. Images analyzed during 1–7 d incubation (Figures 6 and S11–S14) showed growth of cell numbers and few dead cells (red dots) in all groups, indicating the indirect (noncontact) cytocompatibility of the fibers.

## 4 Discussion

The programmed fiber composites showed cell-triggered shape recovery and cytocompatibility when cultured with HepG2 cells, thereby providing demonstration of a cytocompatible SMP that can respond directly to the presence of viable cells via a trigger that is cellular in origin. Shape recovery was successfully triggered by heparin-treated cells in both random and aligned PCL–PEL fibers, with random oriented fibers observed to undergo greater shape change than aligned fibers.

Relative to nontreated cells, heparin-treated cells showed an increase in both intra- and extracellular lipase concentrations, which could explain why shape change was observed in that treatment group, while no significant differences in shape change were observed in non-heparin groups. Addition of heparin has been reported to produce as great as a sevenfold increase in lipase concentrations in HepG2 cells.^40^ In this study, we found a 1.5-fold increase in extracellular lipase concentration and 1.2-fold increase in intracellular lipase concentration when compared to cells not treated with heparin at day 7. The lipase concentration at the surface of fibers to which cells were attached was likely somewhere between the measured intracellular and extracellular concentrations. This localization of elevated lipase concentrations could be a key reason for the SMP of the present work having recovered. In our previous report,^34^ the SMP used herein showed enzymatic shape recovery over 7 d when culturing in a cell-free 0.5 mg/mL lipase solution; in contrast, no shape recovery was observed over 7 d when cultured in the presence of non-lipase-secreting C3H/10T1/2 mouse fibroblasts. In the present work, the extracellular and intracellular lipase secreted by HepG2 cells with the addition of heparin was approximately 2 μg/mL and 4.5 mg/mL, respectively. In addition to the high intracellular lipase concentration, we speculate that a combination of effects may have allowed the HepG2 cells (cultured in the presence of heparin) to trigger the shape recovery.^41,42^ In particular, a four-step sequence has been reported for enzymatic degradation:^43^ adsorption of enzyme onto the surface; formation of a transition complex; scission of the specific chain of PCL; and further interaction of the transition complex with other portions of the polymer. In the present work, by having cells growing on the fibers, lipase was likely secreted directly onto the surface of the fibers, which could facilitate the first step of enzymatic degradation. It has also been reported that an acidic environment accelerates the degradation of PCL,^44^ and in the present work, the cell culture medium containing phenol red tended toward yellow over time, suggesting a mildly acidic environment that may have facilitated the cells effectively digesting or otherwise displacing the lower molecular weight PCL.

Having observed that random fibrous webs recovered more strain than aligned fibrous webs, we speculate that this behavior is due to differences in the microstructural orientation. As discussed in our previous work,^34^ diffusion of lipase may be inhibited by Pellethane, with the degradation mechanism being crystallinity loss, concomitant with the softening of the PCL portion. In the present work, SEM (Figures 3, S6, and S7) showed an aligned and dense structure in the fibers of the aligned samples, which may have resulted in less PCL being exposed to lipase than in the random samples, thus hindering the first step of enzyme degradation. In addition, aligned fibers had less nanofibrous web aligned in the direction of strain programming, and, thus, less force would be generated in the direction of recovery than in random samples, which was expected following previous similar findings from our group.^45^

The particles or binders observed in the fiber structure are hypothesized to be cell debris or cells. As there were no cell fixation step before imaging, cells could have been lysed during the drying and sputter-coating process, which may have produced the observed particles. Cells that were not lysed during the process may have remained in the fiber structure and resulted in the observed binder morphology.

HepG2 cells cultured with SMPs remained healthy, showing no significant difference in viability compared to tissue culture polystyrene controls. This finding demonstrates the indirect cytocompatibility of these cell-responsive SMP materials. This finding complements that of our previous report,^34^ which demonstrated direct cytocompatibility by the characterization of cells that were seeded on fibrous composites.

A potential limitation of the material in its current form is the relatively long recovery time, preventing, for example, the material to be used as an implanted stent, which would require a fast recovery after insertion.^46^ Further, we found that the PCL fixing phase did not completely degrade within a 1 month period. However, as the intracellular lipase concentration by non-heparin-treated cells was still above 0.5 mg/mL, which was essential to trigger the shape recovery, it is speculated that there is potential for cells with no heparin treatment to initiate shape recovery over a long time period.

SMP composites that respond directly to the presence of viable cells via a trigger that is cellular in origin are expected to enable new biomaterial strategies. For example, the particular cell-triggered response demonstrated here could be employed in biosensors to detect the pathological lipase secretion in order to monitor the existence of cancer cells, especially hepatic cancer. Alternatively, the SMP could be used as a drug delivery vehicle that targets the liver tumor area, with the degradation of the PCL fixing phase in proximity to the tumor releasing anticancer drugs.^47,48^

## 5 Conclusions

We have reported a cell-responsive SMP. Shape recovery in response to hepatic cells in the presence of heparin was successfully demonstrated. Importantly, the new fibrous SMP composites were found to be cytocompatible. Inspection of the evolution in shape recovery using SEM revealed that the mechanism involved degradation of the PCL fixing phase that led to a transition from interconnected fibers to a discontinuous fiber structure that allowed the Pellethane shape-memory phase to exert its elasticity and return the shape toward its stress-free configuration. We envision the use of the cell-responsive materials and phenomenon in biomedical fields, expanding the range of SMP-triggering mechanism to include biological cells.

## Supporting Information Available

Supplementary standards, characterization data, and data from experimental groups and replicates not reproduced in full in the main text (PDF)

## Notes

The data that support the findings of this study are available from the corresponding author upon reasonable request.

## References

[ref1] Liu C.; Qin H.; Mather P. T. Review of progress in shape-memory polymers. J. Mater. Chem. 2007, 17, 1543–1558. 10.1039/B615954K.

[ref2] Gopinath S.; Adarsh N. N.; Radhakrishnan Nair P.; Mathew S. One-way thermo-responsive shape memory polymer nanocomposite derived from polycaprolactone and polystyrene-block-polybutadiene-block-polystyrene packed with carbon nanofiber. Mater. Today Commun. 2020, 22, 100802 10.1016/j.mtcomm.2019.100802.

[ref3] Chu C.; Xiang Z.; Wang J.; Xie H.; Xiang T.; Zhou S. A near-infrared light-triggered shape-memory polymer for long-time fluorescence imaging in deep tissues. J. Mater. Chem. B 2020, 8, 8061–8070. 10.1039/D0TB01237H. 32781464

[ref4] Gu X.; Mather P. T. Water-triggered shape memory of multiblock thermoplastic polyurethanes (TPUs). RSC Adv. 2013, 3, 15783–15791. 10.1039/C3RA41337C.

[ref5] Lendlein A.; Langer R. Biodegradable, Elastic Shape-Memory Polymers for Potential Biomedical Applications. Science 2002, 296, 1673–1676. 10.1126/science.1066102. 11976407

[ref6] Wischke C.; Neffe A. T.; Steuer S.; Lendlein A. Evaluation of a degradable shape-memory polymer network as matrix for controlled drug release. J. Controlled Release 2009, 138, 243–250. 10.1016/j.jconrel.2009.05.027. 19470395

[ref7] Maitland D. J.; Small W.; Ortega J. M.; Buckley P. R.; Rodriguez J.; Hartman J.; Wilson T. S. Prototype laser-activated shape memory polymer foam device for embolic treatment of aneurysms. J. Biomed. Opt. 2007, 12, 030504 10.1117/1.2743983. 17614707

[ref8] Wang Y.-J.; Jeng U.-S.; Hsu S.-h. Biodegradable Water-Based Polyurethane Shape Memory Elastomers for Bone Tissue Engineering. ACS Biomater. Sci. Eng. 2018, 4, 1397–1406. 10.1021/acsbiomaterials.8b00091. 33418669

[ref9] Zhang Y.; Zhou S.; Chong K. C.; Wang S.; Liu B. Near-infrared light-induced shape memory, self-healable and anti-bacterial elastomers prepared by incorporation of a diketopyrrolopyrrole-based conjugated polymer. Mater. Chem. Front. 2019, 3, 836–841. 10.1039/c9qm00104b.

[ref10] Sun L.; Huang W. M. Thermo/moisture responsive shape-memory polymer for possible surgery/operation inside living cells in future. Mater. Des. 2010, 31, 2684–2689. 10.1016/j.matdes.2009.11.036.

[ref11] Yu X.; Wang L.; Huang M.; Gong T.; Li W.; Cao Y.; Ji D.; Wang P.; Wang J.; Zhou S. A shape memory stent of poly (ε-caprolactone-co-dl-lactide) copolymer for potential treatment of esophageal stenosis. J. Mater. Sci.: Mater. Med. 2012, 23, 581–589. 10.1007/s10856-011-4475-4. 22057969

[ref12] Wan X.; Wei H.; Zhang F.; Liu Y.; Leng J. 3D printing of shape memory poly (d, l-lactide-co-trimethylene carbonate) by direct ink writing for shape-changing structures. J. Appl. Polym. Sci. 2019, 136, 48177 10.1002/app.48177.

[ref13] Delaey J.; Dubruel P.; Van Vlierberghe S. Shape-Memory Polymers for Biomedical Applications. Adv. Funct. Mater. 2020, 30, 1909047 10.1002/adfm.201909047.

[ref14] Watkin N.; Patel P. The diagnosis and management of acquired urethral stricture disease. Surgery 2020, 38, 212 10.1016/j.mpsur.2020.01.015.

[ref15] Naoum G. E.; Salama L.; Niemierko A.; Vieira B. L.; Belkacemi Y.; Colwell A. S.; Winograd J.; Smith B.; Ho A.; Taghian A. G. Single Stage Direct-to-Implant Breast Reconstruction Has Lower Complication Rates Than Tissue Expander and Implant and Comparable Rates to Autologous Reconstruction in Patients Receiving Postmastectomy Radiation. Int. J. Radiat. Oncol., Biol., Phys. 2020, 106, 514 10.1016/j.ijrobp.2019.11.008. 31756414

[ref16] Meng Y.; Jiang J.; Anthamatten M. Body temperature triggered shape-memory polymers with high elastic energy storage capacity. J. Polym. Sci., Part B: Polym. Phys. 2016, 54, 1397–1404. 10.1002/polb.23990.

[ref17] Lai H.-Y.; Wang H.-Q.; Lai J.-C.; Li C.-H. A Self-Healing and Shape Memory Polymer that Functions at Body Temperature. Molecules 2019, 24, 3224 10.3390/molecules24183224. PMC6767172 31487954

[ref18] Le D. M.; Kulangara K.; Adler A. F.; Leong K. W.; Ashby V. S. Dynamic Topographical Control of Mesenchymal Stem Cells by Culture on Responsive Poly(ϵ-caprolactone) Surfaces. Adv. Mater. 2011, 23, 3278–3283. 10.1002/adma.201100821. 21626577 PMC3972817

[ref19] Davis K. A.; Burke K. A.; Mather P. T.; Henderson J. H. Dynamic cell behavior on shape memory polymer substrates. Biomaterials 2011, 32, 2285–2293. 10.1016/j.biomaterials.2010.12.006. 21224032

[ref20] Ebara M.; Uto K.; Idota N.; Hoffman J. M.; Aoyagi T. Shape-Memory Surface with Dynamically Tunable Nano-Geometry Activated by Body Heat. Adv. Mater. 2012, 24, 273–278. 10.1002/adma.201102181. 21954058

[ref21] Chen H.-M.; Wang L.; Zhou S.-B. Recent Progress in Shape Memory Polymers for Biomedical Applications. Chin. J. Polym. Sci. 2018, 36, 905–917. 10.1007/s10118-018-2118-7.

[ref22] Xiao R.; Huang W. M. Heating/Solvent Responsive Shape-Memory Polymers for Implant Biomedical Devices in Minimally Invasive Surgery: Current Status and Challenge. Macromol. Biosci. 2020, 20, 2000108 10.1002/mabi.202000108. 32567193

[ref23] Yang B.; Huang W. M.; Li C.; Li L. Effects of moisture on the thermomechanical properties of a polyurethane shape memory polymer. Polym. 2006, 47, 1348–1356. 10.1016/j.polymer.2005.12.051.

[ref24] Chen S.; Hu J.; Yuen C.-w.; Chan L. Novel moisture-sensitive shape memory polyurethanes containing pyridine moieties, Polymer (Guildf). Polymer 2009, 50, 4424–4428. 10.1016/j.polymer.2009.07.031.

[ref25] Rochette J. M.; Ashby V. S. Photoresponsive Polyesters for Tailorable Shape Memory Biomaterials. Macromolecules 2013, 46, 2134–2140. 10.1021/ma302354a.

[ref26] Pilate F.; Toncheva A.; Dubois P.; Raquez J.-M. Shape-memory polymers for multiple applications in the materials world. Eur. Polym. J. 2016, 80, 268–294. 10.1016/j.eurpolymj.2016.05.004.

[ref27] Jiang H. Y.; Kelch S.; Lendlein A. Polymers Move in Response to Light. Adv. Mater. 2006, 18, 1471–1475. 10.1002/adma.200502266.

[ref28] Li Z.; Zhang X.; Wang S.; Yang Y.; Qin B.; Wang K.; Xie T.; Wei Y.; Ji Y. Polydopamine coated shape memory polymer: enabling light triggered shape recovery, light controlled shape reprogramming and surface functionalization. Chem. Sci. 2016, 7, 4741–4747. 10.1039/C6SC00584E. 30155125 PMC6014076

[ref29] Wang J.; Brasch M. E.; Baker R. M.; Tseng L.-F.; Peña A. N.; Henderson J. H. Shape memory activation can affect cell seeding of shape memory polymer scaffolds designed for tissue engineering and regenerative medicine. J. Mater. Sci.: Mater. Med. 2017, 28, 151 10.1007/s10856-017-5962-z. 28861660

[ref30] Ebara M.; Akimoto M.; Uto K.; Shiba K.; Yoshikawa G.; Aoyagi T. Focus on the interlude between topographic transition and cell response on shape-memory surfaces. Polym. 2014, 55, 5961–5968. 10.1016/j.polymer.2014.09.009.

[ref31] Chen C.; Hu J.; Huang H.; Zhu Y.; Qin T. Design of a Smart Nerve Conduit Based on a Shape-Memory Polymer. Adv. Mater. Technol. 2016, 1, 1600015 10.1002/admt.201600015.

[ref32] Guo Y.; Lv Z.; Huo Y.; Sun L.; Chen S.; Liu Z.; He C.; Bi X.; Fan X.; You Z. A biodegradable functional water-responsive shape memory polymer for biomedical applications. J. Mater. Chem. B 2019, 7, 123–132. 10.1039/C8TB02462F. 32254956

[ref33] Shou Q.; Uto K.; Lin W.-C.; Aoyagi T.; Ebara M. Near-Infrared-Irradiation-Induced Remote Activation of Surface Shape-Memory to Direct Cell Orientations. Macromol. Chem. Phys. 2014, 215, 2473–2481. 10.1002/macp.201400353.

[ref34] Buffington S. L.; Paul J. E.; Ali M. M.; Macios M. M.; Mather P. T.; Henderson J. H. Enzymatically triggered shape memory polymers. Acta Biomater. 2019, 84, 88–97. 10.1016/j.actbio.2018.11.031. 30471473

[ref35] Choijilsuren G.; Jhou R.-S.; Chou S.-F.; Chang C.-J.; Yang H.-I.; Chen Y.-Y.; Chuang W.-L.; Yu M.-L.; Shih C. Heparin at physiological concentration can enhance PEG-free in vitro infection with human hepatitis B virus. Sci. Rep. 2017, 7, 14461 10.1038/s41598-017-14573-9. 29089529 PMC5663848

[ref36] Rovati L.; Fabbri P.; Ferrari L.; Pilati F. Plastic Optical Fiber pH Sensor Using a Sol-Gel Sensing Matrix. Fiber Optic Sensors; IntechOpen, 2012.

[ref37] Scheibel D. M.; Gitsov I. Unprecedented Enzymatic Synthesis of Perfectly Structured Alternating Copolymers via “Green” Reaction Cocatalyzed by Laccase and Lipase Compartmentalized within Supramolecular Complexes. Biomacromolecules 2019, 20, 927–936. 10.1021/acs.biomac.8b01567. 30592620

[ref38] Wiśniewski J. R.; Vildhede A.; Norén A.; Artursson P. In-depth quantitative analysis and comparison of the human hepatocyte and hepatoma cell line HepG2 proteomes. J. Proteomics 2016, 136, 234 10.1016/j.jprot.2016.01.016. 26825538

[ref39] Nejad H. B.; Robertson J. M.; Mather P. T. Interwoven polymer composites via dual-electrospinning with shape memory and self-healing properties. MRS Commun. 2015, 5, 211–221. 10.1557/mrc.2015.39.

[ref40] Busch S. J.; Martin G. A.; Barnhart R. L.; Jackson R. L. Heparin induces the expression of hepatic triglyceride lipase in a human hepatoma (HepG2) cell line. J. Biol. Chem. 1989, 264, 9527–9532. 10.1016/s0021-9258(18)60563-0. 2542313

[ref41] Matlaga B. F.; Salthouse T. N. Ultrastructural observations of cells at the interface of a biodegradable polymer: Polyglactin 910. J. Biomed. Mater. Res. 1983, 17, 185–197. 10.1002/jbm.820170115. 6826574

[ref42] Woodward S. C.; Brewer P. S.; Moatamed F.; Schindler A.; Pitt C. G. The intracellular degradation of poly(epsilon-caprolactone). J. Biomed. Mater. Res. 1985, 19, 437–444. 10.1002/jbm.820190408. 4055826

[ref43] Sivalingam G.; Chattopadhyay S.; Madras G. Enzymatic degradation of poly (ε-caprolactone), poly (vinyl acetate) and their blends by lipases. Chem. Eng. Sci. 2003, 58, 2911–2919. 10.1016/S0009-2509(03)00155-6.

[ref44] Bartnikowski M.; Dargaville T. R.; Ivanovski S.; Hutmacher D. W. Degradation mechanisms of polycaprolactone in the context of chemistry, geometry and environment. Prog. Polym. Sci. 2019, 96, 1–20. 10.1016/j.progpolymsci.2019.05.004.

[ref45] Wang J.; Quach A.; Brasch M. E.; Turner C. E.; Henderson J. H. On-command on/off switching of progenitor cell and cancer cell polarized motility and aligned morphology via a cytocompatible shape memory polymer scaffold. Biomaterials 2017, 140, 150–161. 10.1016/j.biomaterials.2017.06.016. 28649015 PMC5577642

[ref46] Venkatraman S. S.; Tan L. P.; Joso J. F. D.; Boey Y. C. F.; Wang X. Biodegradable stents with elastic memory. Biomaterials 2006, 27, 1573 10.1016/j.biomaterials.2005.09.002. 16181673

[ref47] Tarvainen T.; Karjalainen T.; Malin M.; Peräkorpi K.; Tuominen J.; Seppälä J.; Järvinen K. Drug release profiles from and degradation of a novel biodegradable polymer, 2,2-bis(2-oxazoline) linked poly(epsilon -caprolactone). Eur. J. Pharm. Sci. 2002, 16, 323–331. 10.1016/s0928-0987(02)00128-8. 12208463

[ref48] Chang S. H.; Lee H. J.; Park S.; Kim Y.; Jeong B. Fast Degradable Polycaprolactone for Drug Delivery. Biomacromolecules 2018, 19, 2302–2307. 10.1021/acs.biomac.8b00266. 29742350

