# Supplementary material for: Cell-Responsive Shape Memory Polymers
Source: ACS Biomater Sci Eng. 2022 Jun 10;8(7):2960–9. doi: 10.1021/acsbiomaterials.2c00405 (PMC9277583; doi:10.1021/acsbiomaterials.2c00405)
Supplement: Supplementary file 1 — ab2c00405_si_001.pdf [file ab2c00405_si_001.pdf]

Supporting Information: Cell-Responsive Shape Memory Polymers

Junjiang Chen[a,b], Lauren E. Hamilton[a,b], Patrick T. Mather[c], James H. Henderson[a,b,*]

a BioInspired Syracuse: Institute for Material and Living Systems, Syracuse University, Syracuse, NY, 13244

b Department of Biomedical and Chemical Engineering, Syracuse University, Syracuse, NY, 13244

c Department of Chemical Engineering, Penn State University, University Park, PA, 16802

* Correspondence author:

James H. Henderson, Department of Biomedical and Chemical Engineering, Syracuse University, Syracuse, NY, 13244, Email: jhhender@syr.edu, TEL: 315-443-9739, fax: 315-443-7724.

15 Pages, 15 Figures

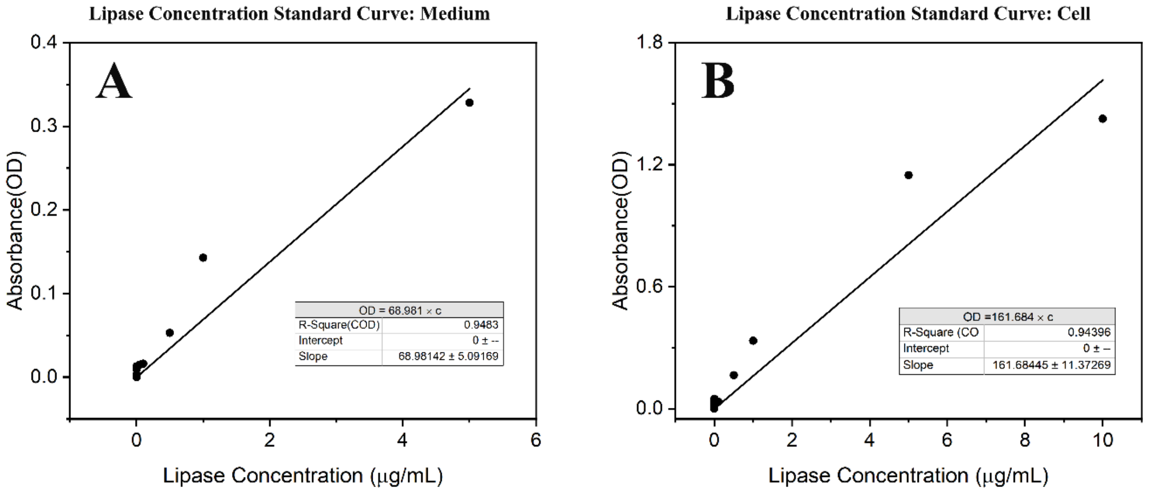

Figure S1. Standard curve for OD vs lipase concentration in the cell culture medium(A) and in the cells (B). Linear regression was performed to fit the standard curve for lipase concentrations A: below 5 μg/mL: OD=68.981c (R^2=0.9483). B: below 10 μg/mL OD= 161.684c (R^2=0.9440).

TGA analysis (Fig. S2) showed a single sharp degradation event for pure PCL in the range from 390 °C to 504 °C, with the maximum decomposition rate occurring at 478 °C. In contrast, Pellethane and PCL-Pellethane showed a two-step degradation. The first degradation of Pellethane occurred in the range from 336 °C to 389 °C, with a 16% weight loss and a maximum decomposition rate occurring at 373 °C, followed by a second degradation at 516 °C, with an additional 65% weight loss and a maximum decomposition rate occurring at 489 °C. The PCL-Pellethane showed a similar degradation range, with 19% and 68% weight losses and maximum decomposition rates occurring at 381 °C and 486 °C, respectively.

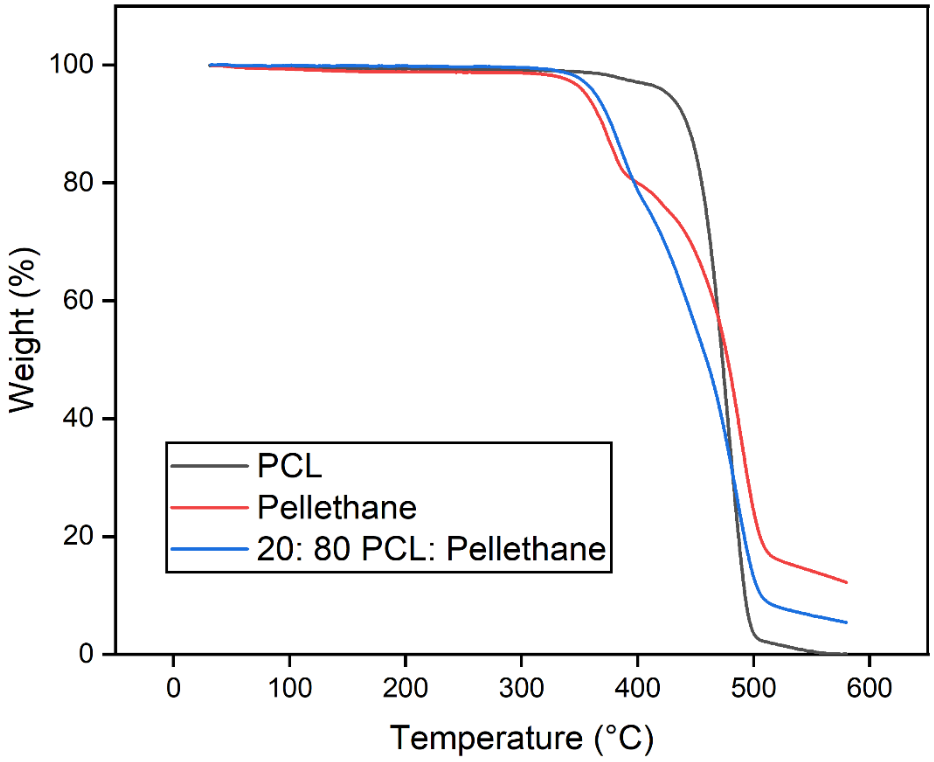

Figure S2. The as-processed fiber composites comprised separate phases as revealed by TGA. Pure PCL showed a single sharp degradation event, whereas Pellethane and PCL-Pellethane showed a two-step degradation.

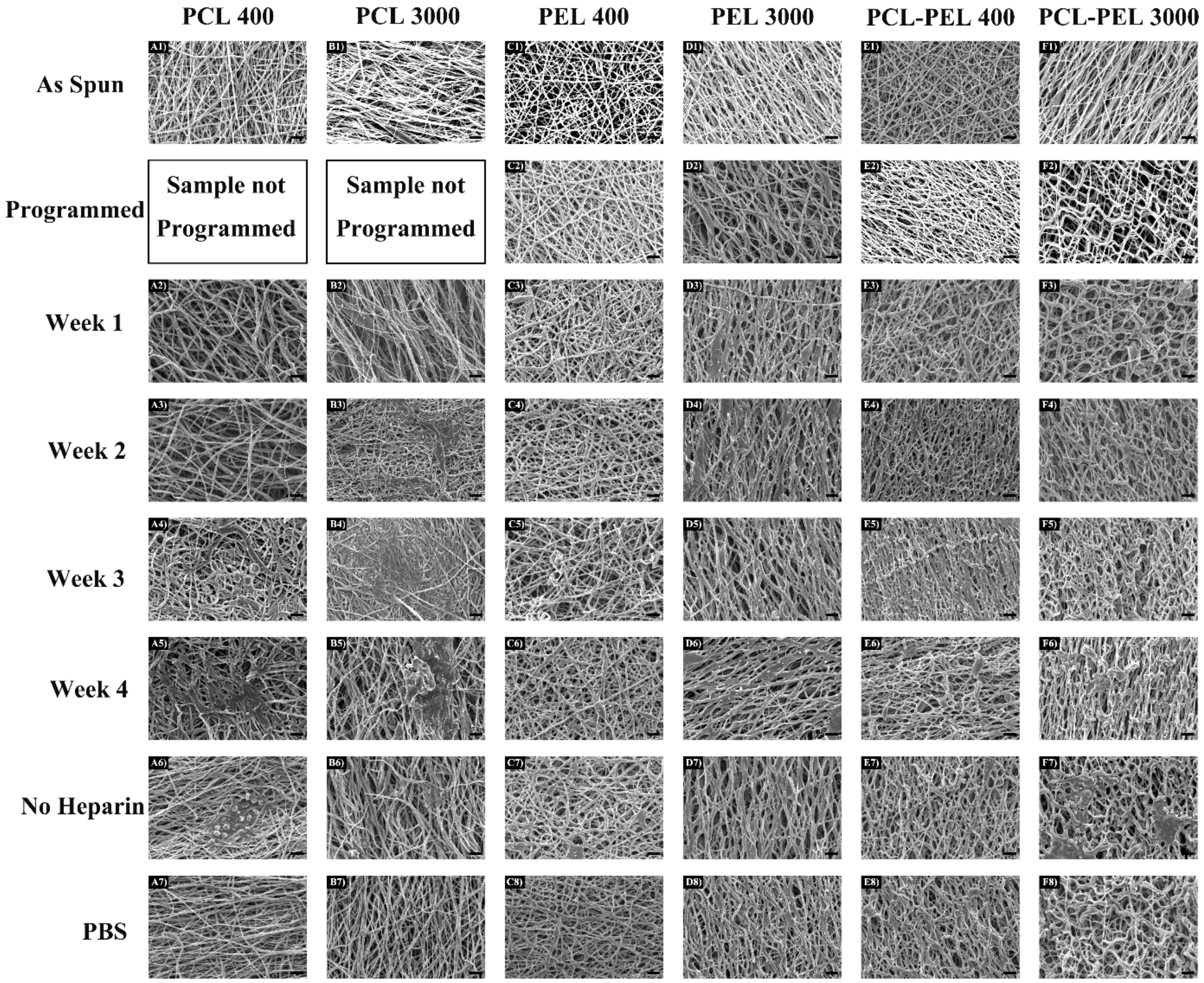

Figure S3. SEM micrographs of all fibers incubated with cells supplied with Heparin for 4 w. All 400 rpm rotation speed electrospun fibers showed a random orientation while 3000 rpm ones showed an aligned orientation. Scale bar: 10 µm.

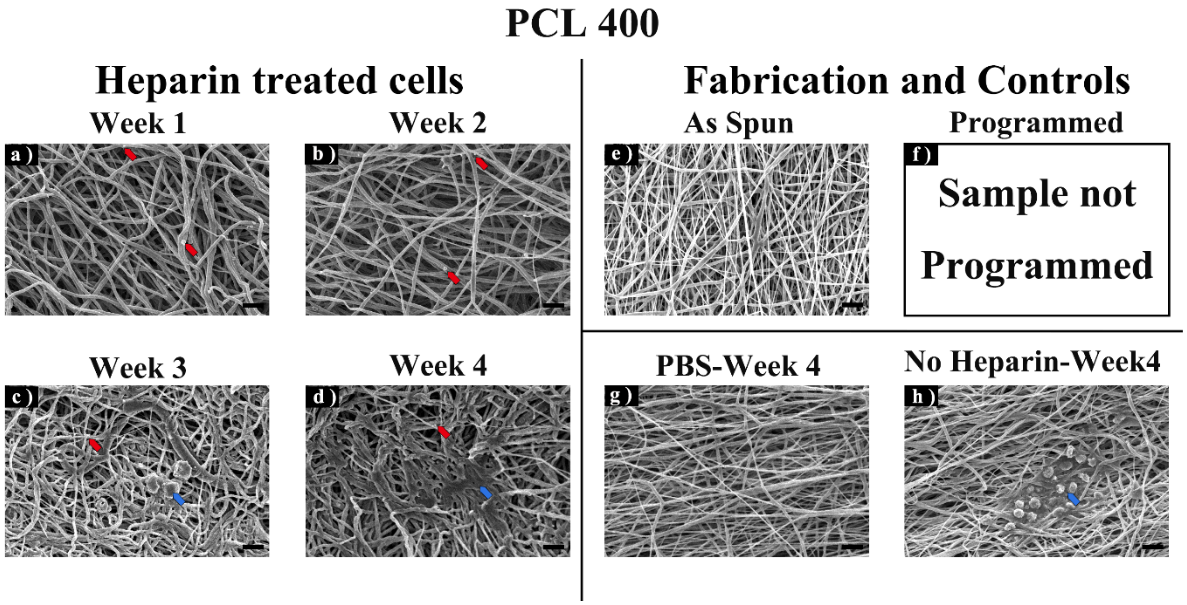

Figure S4. The presence of cells led to a coarser fibers and eventually showed a film-like morphology at 4 w in some areas of the fiber networks, and unidentified small particles or binders (representative particles or binders are identified by arrows) were observed to be embedded in the fiber networks (a, b, c, d, h). Sample cultured with PBS for 4 w showed no morphology change (g). The as spun fibers showed a random orientation (e). Representative particles (red) or binders (blue) are identified by arrows. Scale bar: 10 µm.

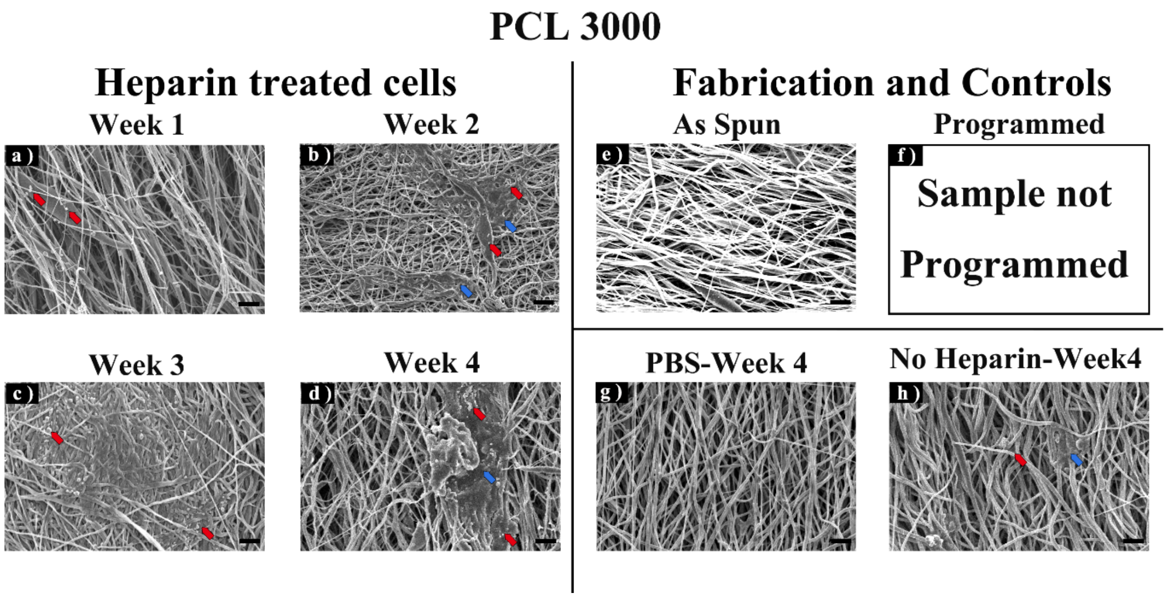

Figure S5. The presence of cells led to coarser fibers and eventually showed a film-like morphology at 4 w in some areas of the fiber networks, and unidentified small particles or binders (representative particles or binders are identified by arrows) were observed to be embedded in the fiber networks (a, b, c, d, h). Sample cultured with PBS for 4 w showed no morphology change (g). The as spun fibers showed an aligned orientation (e). Representative particles (red) or binders (blue) are identified by arrows. Scale bar: 10 µm.

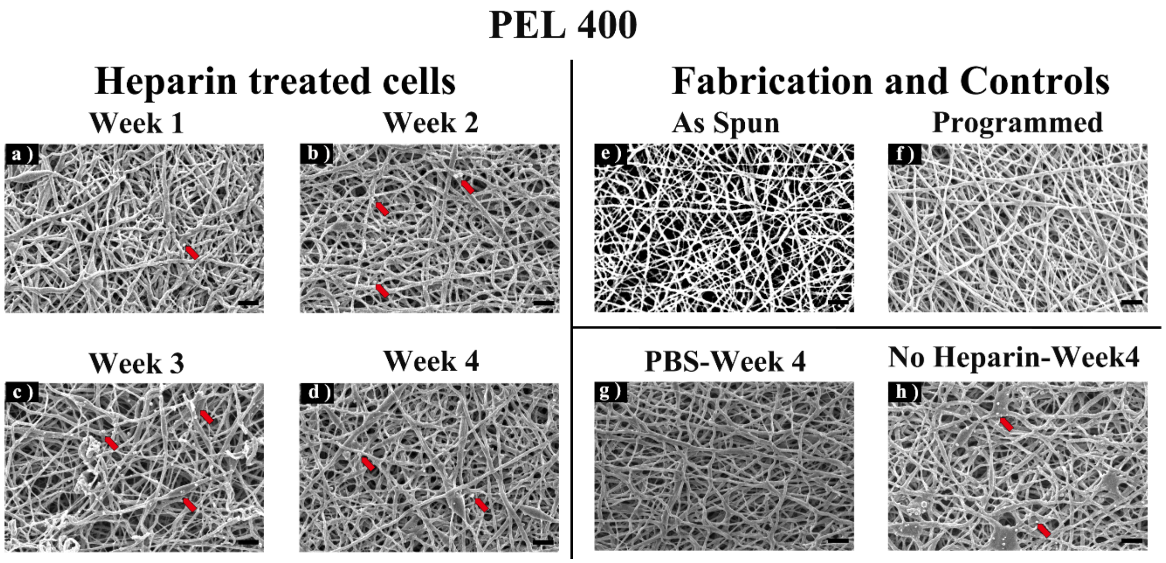

Figure S6. Fibers showed no morphological change after 4 w under any control (g) or treatment condition, and unidentified small particles or binders (representative particles or binders are identified by arrows) were observed to be embedded in the fiber networks with the presence of cells (a, b, c, d, h). The as spun fibers and programmed samples showed a random orientation (e, f). Representative particles (red) or binders (blue) are identified by arrows. Scale bar: 10 µm.

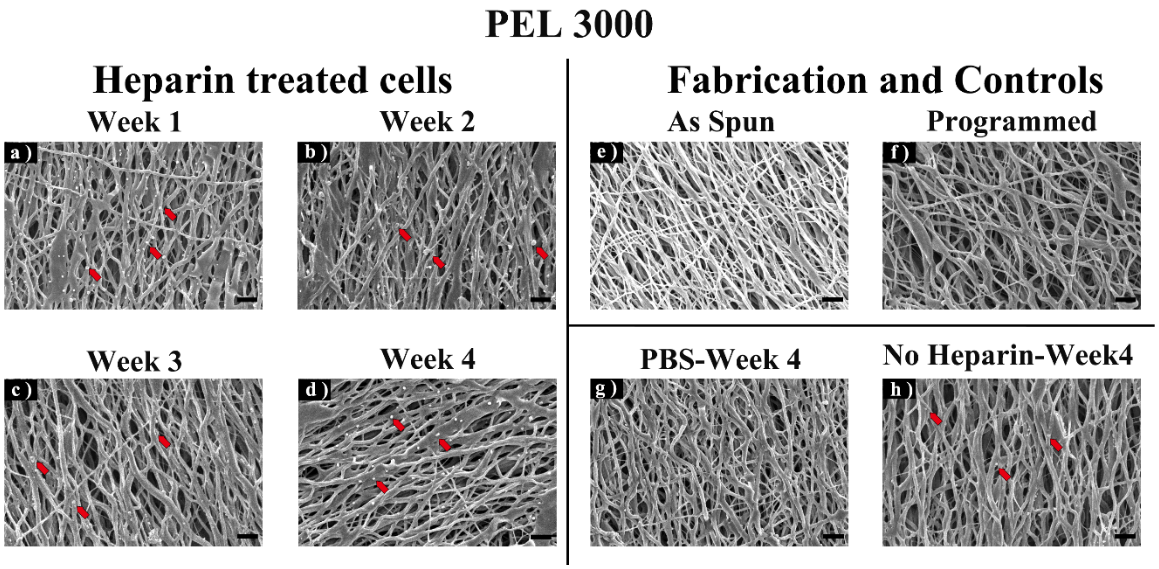

Figure S7. Fibers showed no morphological change after 4 w under any control (g) or treatment condition, and unidentified small particles or binders (representative particles or binders are identified by arrows) were observed to be embedded in the fiber networks with the presence of cells (a, b, c, d, h). The as spun fibers samples showed a random orientation (e), while the programmed one (f) showed a looser arrangement compared to the as-spun samples. Representative particles (red) or binders (blue) are identified by arrows. Scale bar: 10 µm.

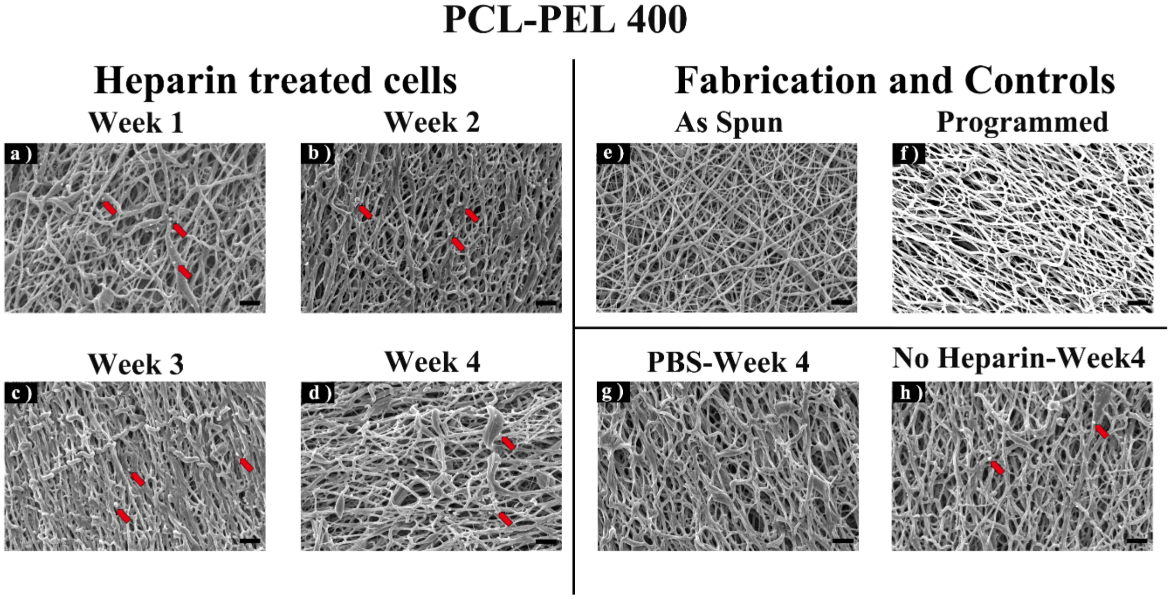

Figure S8. The presence of heparin-treated cells led to recovery of the fiber structure to the as-spun state after 4 w incubation (a, b, c, d), while no significant recovery-related morphological change was found in cells without heparin treatment (h) and PBS (g). Discontinuities of the curling fibers found provide evidence of degradation of PCL over the culture period (a, b, c, d). Unidentified small particles or binders (representative particles or binders are identified by arrows) were observed to be embedded in the fiber networks with the presence of cells (a, b, c, e, h). The as-spun fibers showed a random orientation (e) while the programmed one showed a curved and looser arrangement (f), and after Week 4 fibers (d) showed a similar but looser morphology with more binders and discontinuities among the microstructure compared to the as-spun samples. Representative particles (red) or binders (blue) are identified by arrows. Scale bar: 10 µm.

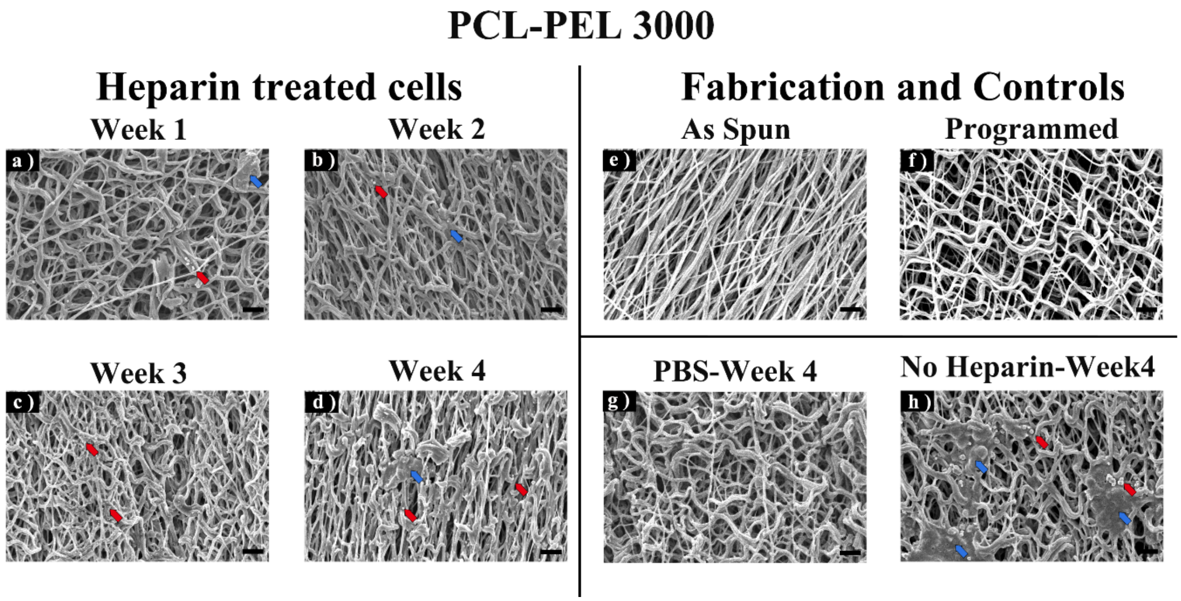

Figure S9. The presence of heparin-treated cells led to recovery of the fiber structure to the as-spun state after 4 w incubation (a, b, c, d), while no significant recovery-related morphological change was found in cells without heparin treatment (h) and PBS (g). Discontinuities of the curling fibers found provide evidence of degradation of PCL over the culture period (a, b, c, d). Unidentified small particles or binders (representative particles or binders are identified by arrows) were observed to be embedded in the fiber networks with the presence of cells (a, b, c, e, h). After 4-week incubation with Heparin treated cells, the fibers (d) showed a similar but looser morphology with more binders and discontinuities among the microstructure compared to the as-spun fibers which showed an aligned orientation (e) and showed a linear and denser structure compared to the programmed one which showed a curved and looser arrangement (f). Representative particles (red) or binders (blue) are identified by arrows. Scale bar: 10 µm.

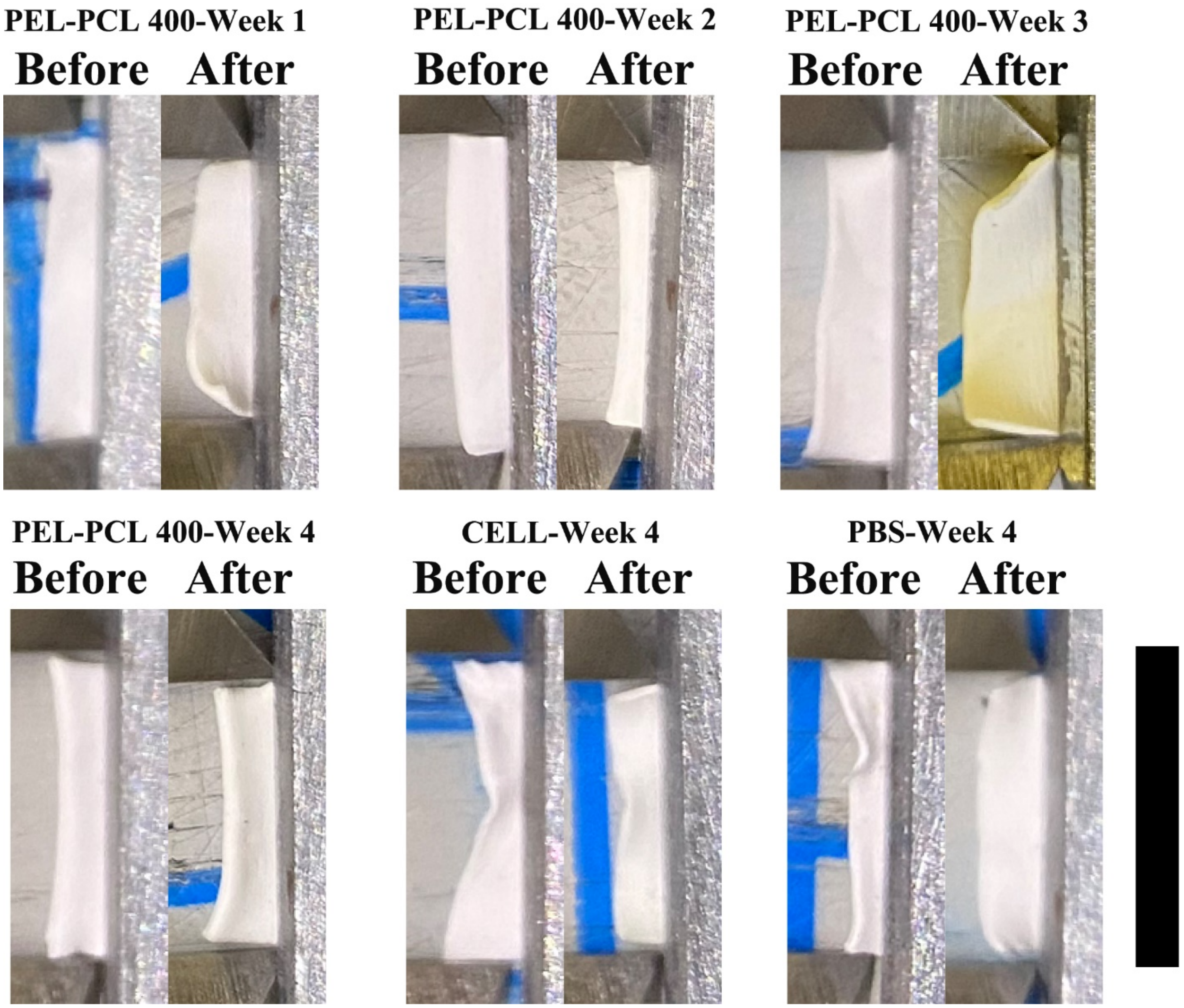

Fig. S10. Macroscopic view of cellular recovery revealed a length change in the sample cultured with HepG2 cells while no length change was observed when samples were cultured in PBS for 4 w. Pictures of samples were taken before and after being exposed to HepG2 cells over the course of 4 w. PEL-PCL 400 samples are shown. Scale bar: 10 mm.

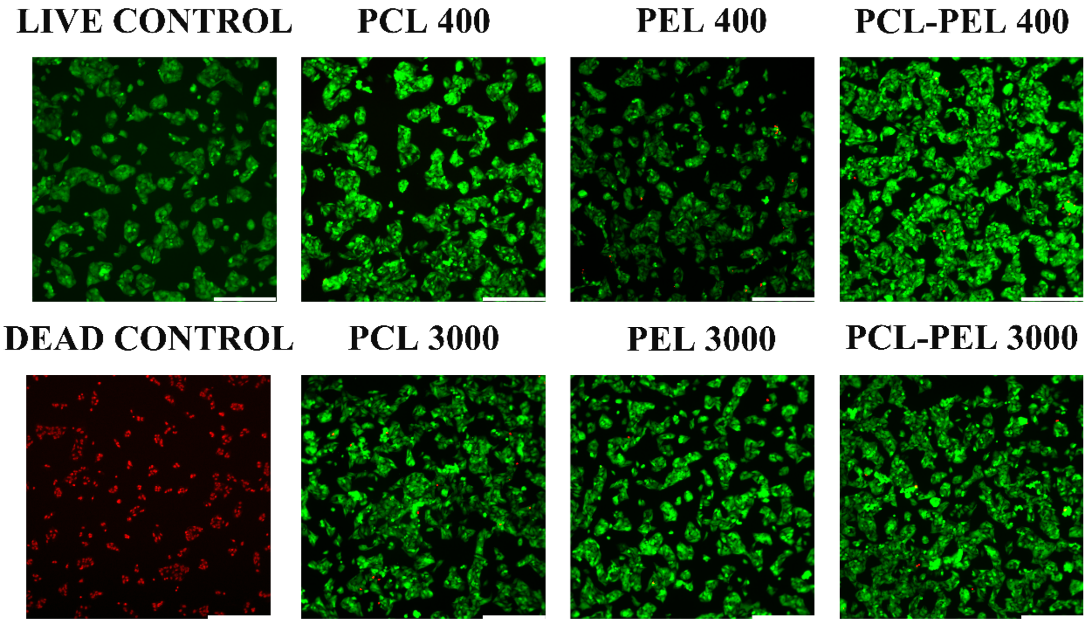

Figure S11. Live/dead micrographs of HepG2 cells cultured on tissue culture plate with fiber composite and non-composite controls for 1 d. Scale bar: 330 µm.

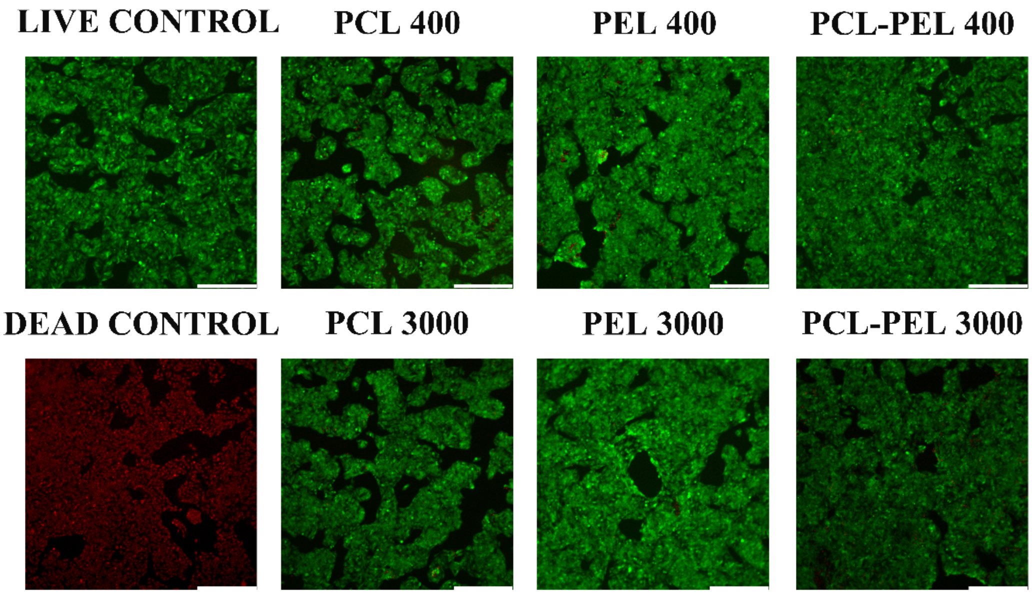

Figure S12. Live/dead micrographs of HepG2 cells cultured on tissue culture plate with fiber composite and non-composite controls for 3 d. Scale bar: 330 µm.

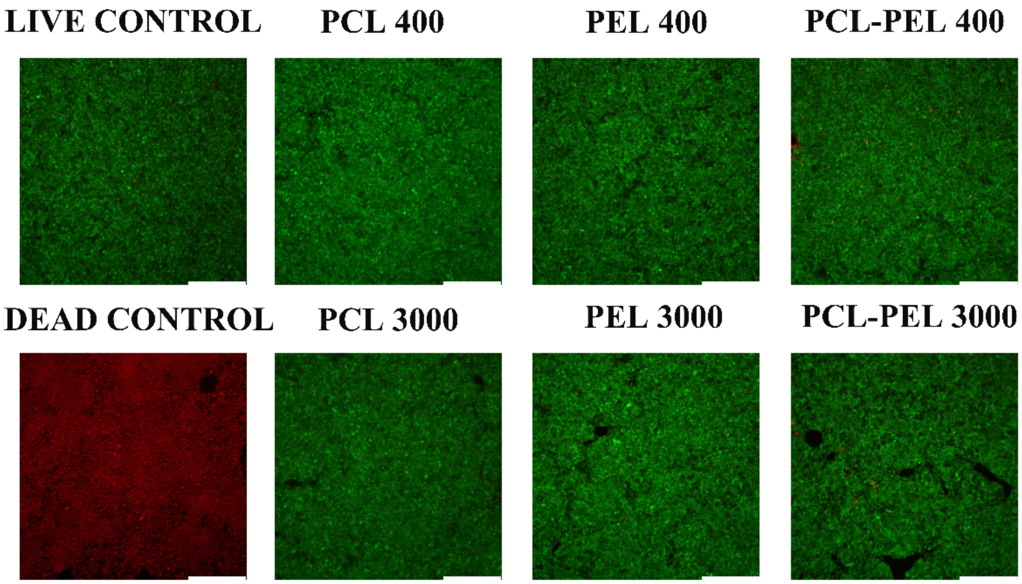

Figure S13. Live/dead micrographs of HepG2 cells cultured directly on tissue culture plate with fiber composite and non-composite controls for 5 d. Scale bar: 330 µm.

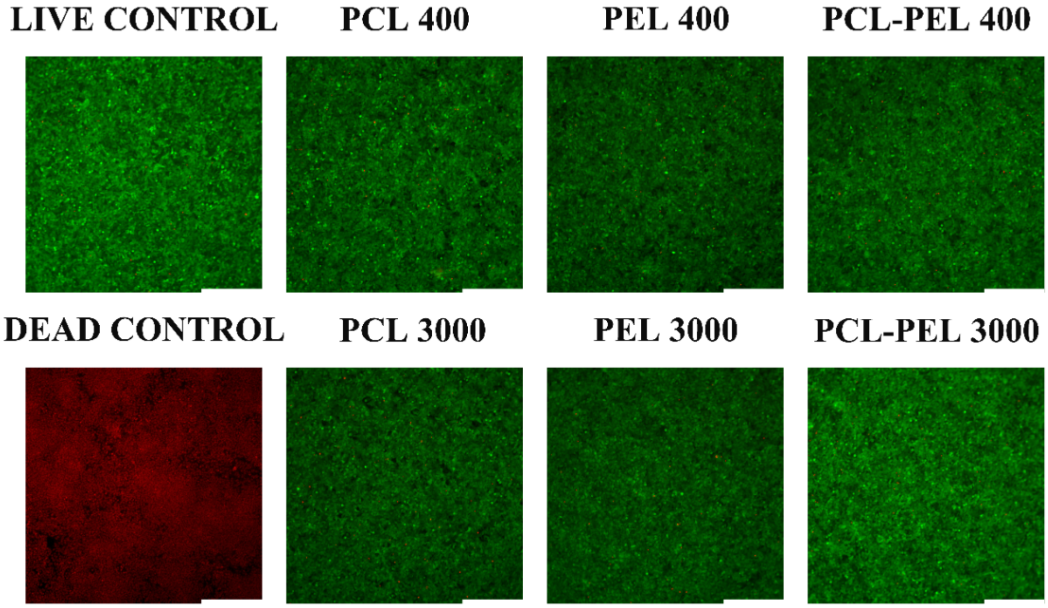

Figure S14. Live/dead micrographs of HepG2 cells cultured on tissue culture plate with fiber composite and non-composite controls for 7 d. Scale bar: 330 µm.
